# Quality Social Connection as an Active Ingredient in Digital Interventions for Young People With Depression and Anxiety: Systematic Scoping Review and Meta-analysis

**DOI:** 10.2196/26584

**Published:** 2021-12-17

**Authors:** Lindsay H Dewa, Emma Lawrance, Lily Roberts, Ellie Brooks-Hall, Hutan Ashrafian, Gianluca Fontana, Paul Aylin

**Affiliations:** 1 Institute of Global Health Innovation Imperial College London London United Kingdom; 2 School of Public Health Imperial College London London United Kingdom; 3 Mental Health Innovations London United Kingdom

**Keywords:** mental health, digital interventions, young people, quality social connection, depression, anxiety, systematic review, meta-analysis, patient and public involvement, mobile phone

## Abstract

**Background:**

Disrupted social connections may negatively affect youth mental health. In contrast, sustained quality social connections (QSCs) can improve mental health outcomes. However, few studies have examined how these quality connections affect depression and anxiety outcomes within digital interventions, and conceptualization is limited.

**Objective:**

The aim of this study is to conceptualize, appraise, and synthesize evidence on QSC within digital interventions (D-QSC) and the impact on depression and anxiety outcomes for young people aged 14-24 years.

**Methods:**

A systematic scoping review and meta-analysis was conducted using the Joanna Briggs Institute methodological frameworks and guided by experts with lived experience. Reporting was guided by the PRISMA (Preferred Reporting Items for Systematic Reviews and Meta-Analyses). The MEDLINE, Embase, PsycINFO, and CINAHL databases were searched against a comprehensive combination of key concepts on June 24, 2020. The search concepts included young people, digital intervention, depression, anxiety, and social connection. Google was also searched. A reviewer independently screened abstracts and titles and full text, and 9.99% (388/3882) of these were screened by a second reviewer. A narrative synthesis was used to structure the findings on indicators of D-QSC and mechanisms that facilitate the connection. Indicators of D-QSC from the included studies were synthesized to produce a conceptual framework.

**Results:**

Of the 5715 publications identified, 42 (0.73%) were included. Among the included studies, there were 23,319 participants. Indicators that D-QSC was present varied and included relatedness, having a sense of belonging, and connecting to similar people. However, despite the variation, most of the indicators were associated with improved outcomes for depression and anxiety. Negative interactions, loneliness, and feeling ignored indicated that D-QSC was not present. In 24% (10/42) of the applicable studies, a meta-analysis showed a significant decrease in depression (–25.6%, 95% CI –0.352 to –0.160; *P*<.001) and anxiety (–15.1%, 95% CI –0.251 to –0.051; *P*=.003) after a D-QSC. Digital mechanisms that helped create a quality connection included anonymity, confidentiality, and peer support. In contrast, mechanisms that hindered the connection included disconnection from the real world and inability to see body language. Data synthesis also identified a 5-component conceptual framework of D-QSC that included rapport, identity and commonality, valued interpersonal dynamic, engagement, and responded to and accepted.

**Conclusions:**

D-QSC is an important and underconsidered component for youth depression and anxiety outcomes. Researchers and developers should consider targeting improved QSC between clinicians and young people within digital interventions for depression. Future research should build on our framework to further examine relationships among individual attributes of QSC, various digital interventions, and different populations.

## Introduction

### Background

Enforced lockdowns and physical distancing measures introduced to slow the COVID-19 infection rate have resulted in disrupted face-to-face connections. Ordinarily, a lack of meaningful social connections through social isolation is associated with poor health outcomes such as sleep problems, loneliness, depression, and anxiety, leading in some cases to suicide. Young people are particularly vulnerable to mental health difficulties such as depression and anxiety because onset usually occurs before the age of 24 years [1], and they are often comorbid globally [2]. Although disrupted social connections and loneliness can have a negative effect on mental health [3,4], feeling socially connected is one of the strongest protective factors for depression [5] and can decrease symptoms of anxiety [6].

Social connection as a concept is multifaceted. It can be described as the quantity of connections, the opposite of loneliness, or as having social support. In our context, social connection is the perceived value of attributes of a meaningful interaction among 2 or more people or a *quality* social connection (QSC). Such valued attributes can include, for example, feeling listened to, understood, and a sense of belonging. Similarly, a cooperative relationship between client and therapist, comprising a close bond, shared goals, and tasks, is defined as a *therapeutic alliance* in face-to-face therapy [7]. A therapeutic alliance has been shown to significantly modulate treatment outcomes [8], including in digital settings [9]. Similarly, *social prescribing* to improve social connection has decreased loneliness and improved health outcomes [10]. However, studies have only subjectively demonstrated the value of strong social networks and social relationships for both physical and mental health [11] and longevity [12]. This suggests a need for well-defined indicators of social connection to enable objective measurement of these effects.

The COVID-19 pandemic has accelerated a rapid shift to digital provision of formal and informal mental health support [13]. Indeed, mental health care is often seen as the best candidate for a *digital revolution* because prevention and treatment, including talking therapies, are amenable to delivery over screens and remotely [14]. Social media, video consultations, texting, and virtual reality are interventions that can enable social connections [13]. They represent an important intervention for young people with mental health difficulties to strengthen new and existing relationships and facilitate peer-to-peer and formal mental health support [15]. However, digital interventions such as social media use are also associated with negative consequences such as cyberbullying, viewing harmful content, and a greater sense of isolation [16]. This contradiction requires further investigation to identify the ways in which digital interventions may help or hinder QSC.

Young people are the most digitally fluent and most in need of mental health support. However, QSC within digital interventions (D-QSC) has received little attention in relation to outcomes for depression and anxiety in young people. A recent review produced a conceptual framework for *social connectedness* but positioned it as a solution to loneliness and not as an *active ingredient* (best bet) for the prevention and treatment of depression and anxiety [17]. It also did not consider digital interventions or young people. A systematic review is needed to help produce a conceptual framework for indicators of D-QSC that can be applied to examine its influence on depression and anxiety outcomes across contexts. Our study aims are to (1) identify indicators of D-QSC and their ability to improve or worsen outcomes for depression and anxiety in young people across contexts, (2) identify digital intervention mechanisms that facilitate QSC, and (3) produce a conceptual framework for indicators of D-QSC.

### Research Questions

The research questions are:

1. What indicates the presence of QSC in nondigital and digital interventions?
2. How does D-QSC improve or worsen outcomes for depression and anxiety in young people?
3. What digital intervention mechanisms facilitate QSC when preventing or treating depression and anxiety in young people?
4. Whom does D-QSC help or hinder across different contexts, user preferences, and levels of engagement?

## Methods

### Design

#### Overview

This systematic scoping review was conducted using the Joanna Briggs Institute methodological framework for scoping reviews. Reporting was guided by the PRISMA (Preferred Reporting Items for Systematic Reviews and Meta-Analyses; Multimedia Appendix 1) guidelines to ensure clear structure, reproducibility, and rigor.

#### Defining Objectives and Questions and Developing Inclusion Criteria

The research questions were considered, refined, and then finalized with all team members. The Population, Intervention, Comparison, Outcomes, and Study Design Tool was used to produce our inclusion and exclusion criteria (Table 1). Notably, *young people* (population) as a definition is heterogeneous. However, we have chosen the age group of 14-24 years because it captures key points of vulnerability to developing anxiety and depression between midadolescence and emerging adulthood.

**Table 1:** Selection criteria.

Category	Inclusion criteria	Exclusion criteria
Population	Young people aged 14-24 years Young people aged 14-24 years and additionally 1 year either side of this range (eg, young people aged 13-16 years would be included, whereas those aged 16-26 years would be excluded)	Nonhuman subjects Adults aged ≥25 years if unable to easily separate results from those of younger group
Intervention	Explores QSC^a^ (ie, mentions relevant attributes such as empathy, feeling listened to, understood by another person) Use of a digital intervention, software, or internet-delivered services (eg, smartphone app, virtual reality packages, internet-based treatment, and chat room)	No mention of QSC (eg, focuses only on quantity of connections) No mention of digital intervention (eg, based on a face-to-face situation only)
Comparator	N/A^b^	N/A
Outcome	Scope of depression and anxiety spanned all forms, including major, bipolar, psychotic, perinatal, postpartum, PMDD^c^, and manic depression, as well as social, generalized, OCD^d^, panic, PTSD^e^, and anxiety disorders Influence on existing symptoms of depression or anxiety (eg, mood and self-esteem through self-report questionnaire or clinical interview) Prevention of onset of depression or anxiety (eg, measuring mental well-being through self-report questionnaire or clinical interview)	No mention of depression or anxiety No mention of the influence on existing symptoms of depression or anxiety No mention of the influence on depression or anxiety prevention
Study design	All study designs	N/A
Dates	From earliest date to June 24, 2020	Outside date remit

^a^QSC: quality social connection.

^b^N/A: not applicable.

^c^PMDD: premenstrual dysphoric disorder.

^d^OCD: obsessive-compulsive disorder.

^e^PTSD: posttraumatic stress disorder.

#### Searching for the Evidence

The MEDLINE, Embase, PsycINFO, and CINAHL databases were searched on June 24, 2020. The search strategy was developed and verified by 3 team members (LD, EL, and HA) and an institutional librarian and tailored to each database (Multimedia Appendix 2 [18-59]). In all, 4 facets made up the strategy, including young people (eg, *youth* and *teens*), social connection (social connect* and *sociali?ation*), digital intervention (eg, *online* and *digital*), and depression and anxiety (depress* and anx*). The World Health Organization International Clinical Trials Registry Platform, ClinicalTrials.gov, and the *Journal of Medical Internet Research* were searched on July 14, 2020. The first 100 Google search hits were also systematically searched by 2 reviewers (LR and EBH) using key words across the 4 facets (eg, young people, social connect*, anxiety and depression, and digital) as a further check (Multimedia Appendix 2). The included papers’ reference lists were also reviewed and added to the search if appropriate.

#### Selecting the Evidence

Titles and abstracts were independently screened by 1 reviewer (LR) and excluded if they did not match the selection criteria (Table 1). Studies that met the inclusion criteria were retrieved in full by the primary reviewer (LR) and reassessed against the selection criteria in detail. A second reviewer (EBH) independently screened a random 9.99% (388/3882) of the titles, abstracts, and full-text manuscripts to ensure reliability in study selection. A predefined interreliability agreement (≥0.70) was agreed upon and calculated. Another random 9.99% (388/3882) would have been screened until agreement was achieved. Disagreements were resolved with a third reviewer (LD).

#### Extracting and Charting the Evidence

The data-charting process documented indicators of QSC, prevention and treatment categorization, digital intervention mechanisms that facilitate QSC, and participant characteristics. An initial 20% (8/42) of the studies were extracted independently by 2 reviewers (LR and EBH) and reviewed to ensure accuracy before 1 reviewer (LR) continued with the remaining extraction. All included studies were also appraised using the Hawker checklist [60], which is designed specifically for cross-comparison across heterogeneous designs (quantitative, qualitative, and mixed methods). A total of 9 domains were appraised: (1) abstract and title, (2) introduction and aims, (3) methods and data, (4) sampling, (5) data analysis, (6) ethics and bias, (7) results, (8) transferability and generalizability, and (9) implications and usefulness. Quality scores were assigned to each domain, from 1 point (very poor) to 4 points (good), summed and assigned as high quality (30-36 points), medium quality (24-29 points), or low quality (9-23 points).

#### Analysis of the Evidence, Presentation of the Results, and Summarizing the Evidence

Meta-analyses were performed where appropriate to examine the effect of D-QSC on outcomes. Overall and specific categories of depression, anxiety, and well-being outcomes were analyzed by calculating the ratio of means within each study. We substituted median for mean in studies where only the median was reported. The inverse-variance, random-effects model of DerSimonian and Laird [61] was used for analysis of both continuous and categorical variables in Stata software (version 15; StataCorp) [62]. The I^2^ statistic was used to estimate the degree of heterogeneity among studies, where larger values indicated increasing heterogeneity. The scoping nature of the review meant that a narrative approach was appropriate. All indicators of the development and presence of D-QSC were first collated and synthesized using a deductive approach. The initial relationship between these indicators and the outcomes was explored. Potential themes were identified, discussed, and agreed upon by 3 reviewers (LD, LR, and EL).

To produce a conceptual framework for indicators of D-QSC there were 4 main stages. At the first stage, all indicators identified in the literature synthesis or by experts with lived experience (see the *Patient and Public Involvement* section) were added as cards in Miro (ie, participative visual platform). Indicators that directly described *social connection* (eg, social connectedness) were repeated and those that were not an attribute of D-QSC were excluded. Second, the remaining indicators were either grouped with similar indicators or stood alone. Third, the indicators were then mapped onto a preexisting framework of the components of social connectedness in mental disorders (closeness, identity and common bond, valued relationships, involvement, and cared for and accepted [CIVIC] framework) [17]. Indicators that did not map onto the preexisting framework were kept together and merged under a new component name. This resulted in preliminary components of D-QSC. Finally, young experts with lived experience critically reflected on the preliminary framework and answered a series of questions at a web-based meeting and through email. For example, “Is any indicator missing?” and “Do the indicators link together well or should they be moved?” This discussion was unstructured to allow independent and novel thought. As a result, changes were made to either component or indicator wording and indicators were added or merged. All team members and the young experts agreed on the final conceptual framework for D-QSC.

### Patient and Public Involvement

We advertised for young people aged 14-24 years with experience of depression or anxiety and digital interventions for mental health to work on a review about social connection in the digital world through The McPin Foundation newsletter, email distribution lists, Twitter, and Instagram. A total of 9 people applied using a simple form, and all joined the Young Persons Advisory Group (YPAG). They represented different genders, ethnicities, ages, and UK locations. We held an initial web-based workshop to help define QSC and D-QSC, inform search terms, and review the protocol. At this stage, we approached the Lancet Commission for Global Mental Health Young Leaders and experts with professional experience (ie, delivery of digital interventions) to ensure a diverse range of experiences, cultural contexts (ie, low- and high-resource settings), and experience of youth interventions for depression or anxiety. We had separate discussions with each group on the web (eg, Zoom). Subsequent changes were made to our definition of D-QSC, selection criteria, and protocol. On the basis of definitions of quality [63] and therapeutic alliance [7] and input from team members (EL, LD, and LR) and experts, QSC was then operationally defined as *the* *perceived value of the attributes of an interaction between two or more people*. Key attributes (ie, indicators) of D-QSC that made up the definition were logged across the 3 expert groups and amalgamated with the literature indicators as described previously. Others were changed (eg, changed to plain English) or merged after the YPAG and the Lancet Commission for Global Mental Health Young Leaders were shown the findings and conceptualization framework. A YPAG member (EBH) also screened, extracted, and quality-assessed literature. All were given appropriate support and paid in line with guidance [64].

## Results

### Overview

**Figure 1:**
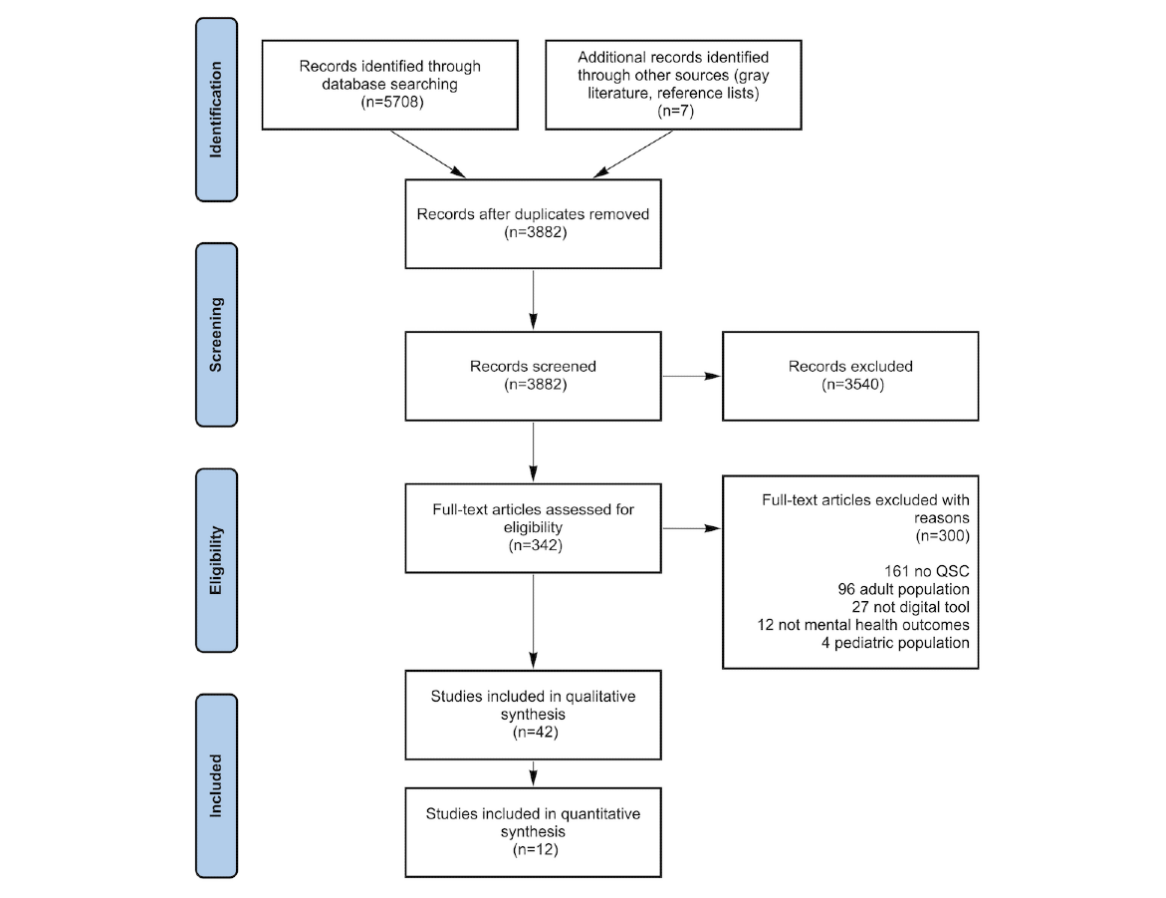
PRISMA (Preferred Reporting Items for Systematic Reviews and Meta-Analyses) flowchart. QSC: quality social connection.

A total of 5715 records were identified (Figure 1). Of the 5715 articles, 1833 (32.07%) were duplicates and were removed. Substantial agreements were achieved in the screening of the random 9.99% (388/3882) abstracts and titles as well as full-text subsamples (K=0.80 and K=0.70, respectively). Papers were then excluded if they did not match the selection criteria; of the 3882 publications remaining after duplicates were removed, 42 (1.08%) were included (Figure 1). Of these 42 studies, 28 (67%) were of high quality, 13 (31%) were of medium quality, and 1 (2%) was of low quality. High-quality studies largely demonstrated good explanation of aims, methods, and sampling to enable replicability. Medium-quality studies included most of the good study criteria but were lacking in some areas, which reduced their scores. The low-quality study did not provide enough detail across most domains (eg, it did not report ethical considerations, the results were unclear, and the methods were not replicable).

### Study and Participant Characteristics

Of the 42 included studies, 25 (60%) were quantitative [18-42], 11 (26%) were qualitative [43-53], and 6 (14%) were mixed methods studies [54-59] (Table 2; Multimedia Appendix 3 [18-59]). The studies mainly used uncontrolled or cross-sectional designs and had questionnaires as their main data collection method (22/42, 52%). All studies took place in high-income countries: the United States [23-28,36,38,40,42,44,47,49,50,53], Australia [22,29-31,43,46,55-58], Ireland [18,45,54], Israel [19,32,48], Taiwan [20,35], Sweden [21,41], the Netherlands [33], Turkey [39], Austria [34], Belgium [37], Canada [59], Cyprus [52], and the United Kingdom [51]. There were 23,319 participants in total (12,825/23,319, 55%, were women; of the 42 studies, 2 (5%) did not report participant gender). Of the 42 studies, 21 (50%) focused on both prevention and treatment [18,20,21,23-29,36-39,41-44,48,50,57], 12 (29%) focused on treatment [22,30,31,45,49,51-56,58], and 9 (21%) focused on prevention [19,32-35,40,46,47,59]. Digital mental health–related intervention types included mental health social networking tools, smartphone apps, self-help cognitive behavioral therapy, telepsychiatry, one-to-one peer mentor support, video gaming, avatars, and internet use for mental health support. Nonspecific informal digital interventions included general social networking and social media (eg, Facebook, Twitter, Tumblr, Snapchat, and Reddit) and general internet use and web browsing. Intervention duration was reported in 48% (20/42) of the studies and ranged from 8 weeks to 1 year.

**Table 2:** Data extraction and quality assessment of included studies (N=42).

Author, year, country, quality	Study design	Setting and participants	Digital intervention	Outcomes and measures
Alvarez-Jimenez et al [58], 2013, Australia, high quality	Quantitative and qualitative, uncontrolled single-group, observation, questionnaire, and semistructured interview	Setting: early psychosis prevention and intervention center; sample: 20 patients (50% female; aged 15-25 years; 45% Anglo-Australian, 25% Asian, 10% biracial, and 5% African); presenting condition: first episode psychosis	Peer-to-peer web-based social networking, individually tailored web-based psychosocial interventions, and expert moderation: HORYZONS	Outcomes: depression and anxiety reduced; measures: BPRS^a^, CDRS^b^, and BAI^c^
Alvarez-Jimenez et al [30], 2018, Australia, medium quality	Quantitative, uncontrolled single-group, observation, and semistructured interview	Setting: PACE^d^ clinic for ultrahigh-risk psychosis; sample: 14 patients (79% female; aged 15-25 years; ethnicity unknown; all Australia-born); presenting condition: ultrahigh risk for psychosis	Web-based social networking, peer-to-peer and professional moderation: MOMENTUM	Outcomes: depression reduced and psychological well-being improved; measures: SWLS^e^, MADRS^f^, and PSSS^g^
Bailey et al [22], 2020, Australia, high quality	Quantitative, uncontrolled single-group pre- and posttest, observation, and semistructured interview	Setting: tertiary-level mental health service; sample: 20 patients (55% female; aged 16-25 years; ethnicity unknown; country of birth: 75% Australia, 20% Asia, and 5% United Kingdom); presenting condition: suicidal ideation	Enhanced web-based social networking intervention: Affinity	Outcome: depression reduced; measure: PHQ-9^h^
Bhuvaneswar and Gutheil [49], 2008, United States, high quality	Qualitative, retrospective case study, and observation	Setting: psychodynamic psychotherapy clinic; sample: 1 patient (female; aged 17 years; ethnicity unknown); presenting condition: depression	Instant messenger	Outcome: psychological well-being worsened; measure: self-report
Blackwell et al [24], 2012, United States, high quality	Quantitative, randomized controlled trial, and questionnaire	Setting: general; sample: 100 adolescents (62% female; mean age 15.69 years, SD 2.91 years; 57% White, 16% Hispanic, 9% African American, and 18% ethnicity unknown); presenting condition: cystic fibrosis	Web-based social networking peer support program: CFfone.com	Outcomes: Depression and anxiety reduced; measure: HADS^i^
Campbell et al [55], 2019, Australia, medium quality	Qualitative and quantitative, participatory action research design, observation, and questionnaire	Setting: Kids Helpline family discord service; sample: 105 callers to helpline (82% female; aged 13-25 years; ethnicity unknown); presenting condition: mild to moderate depression or anxiety (not high risk)	Social networking site for peer-to-peer and counsellor-to-peer group support	Outcomes: depression and anxiety—data quality too low to assess; measures: CES-D^j^ and RCMAS^k^
Canady [25], 2018, United States, high quality	Quantitative, cross-sectional study, questionnaire, and interview	Setting: general; sample: 1300 adolescents (gender unknown; aged 14-22 years; ethnicity unknown); presenting condition: none in particular	Web-based health information and digital health tools in general, including peer-to-peer health exchange networks	Outcomes: Depression and anxiety reduced; measures: PHQ-9 and self-report
Chyzzy et al [59], 2020, Canada, high quality	Qualitative and quantitative, uncontrolled single-group design, questionnaire, and semistructured interview	Setting: MPPS^l^ intervention group; sample: 21 mothers (100% female; aged 17-24 years, mean age 21.3, SD 1.8, years; ethnicity unknown; country of birth: 66.7% Canada), presenting condition: generally healthy, 14.3% with prior history of depression	Individualized peer mentor support through telephone call and SMS text messaging: MPPS intervention	Outcomes: depression and anxiety reduced; measure: self-report
Clarke [45], 2018, Ireland, high quality	Qualitative, retrospective case study, and observation	Setting: clinical; sample: 1 patient (male; aged 16 years; ethnicity unknown); presenting condition: Asperger syndrome with comorbid depression	Telepsychiatry	Outcome: depression treatment engagement improved; measure: observation
Colder Carras et al [28], 2017, United States, medium quality	Quantitative, cross-sectional study, and questionnaire	Setting: 30 US schools; sample: 9733 students (51% female; aged 13-16 years, average age 14.1 years; 82.1% Dutch); presenting condition: none in particular	Web-based video gaming	Outcomes: depression and social anxiety reduced for *social engaged gamers* compared with *problematic*, *at-risk,* or *extensive* gamers; measures: depressive mood list and SASC-R^m^
Cole et al [36], 2017, United States, —^n^	Quantitative, uncontrolled single-group design, and questionnaire	Setting: private university; sample: 231 undergraduate students (72% female; average age 19.28, SD 1.15, years; 67.1% White, 23.4% Asian American, 10.4% African American, 5.2% Hispanic or Latino, and 0.4% Other); presenting condition: none in particular	Web-based social networks in general	Outcomes: depression worsened; measures: DASS^o^, CTI^p^, and BDI-II^q^
Dhesi [51], 2019, United Kingdom, high quality	Qualitative, cross-sectional, and web-based semistructured interviews	Setting: Kooth digital mental health care service; sample: 13 Kooth users (69% female; aged 14-18 years; 69.2% White British, 15.4% White and Asian, and 15.4% Other); presenting condition: none in particular	Web-based counseling (text)	Outcomes: anxiety reduced; measure: thematic analysis of interviews
Dolev-Cohen and Barak [48], 2013, Israel, high quality	Qualitative, case-control design, questionnaire, textual analysis, and observation	Setting: general; sample: 150 instant messaging users (63% female; aged 14-18 years; ethnicity unknown); presenting condition: distressed vs nondistressed groups of participants	Regular use of instant messaging	Outcome: psychological well-being improved; measure: PANAS^r^
Ellis et al [56], 2011, Australia, —	Qualitative and quantitative, comparative randomized controlled trial, and questionnaire	Setting: university students not receiving mental health treatment; sample: 39 students (77% female; aged 18-25 years, mean age 19.67, SD 1.66, years; ethnicity unknown); presenting condition: anxiety or depression but none severe	Web-based cognitive behavior therapy self-help program (MoodGYM) compared with web-based support group (MoodGarden)	Outcomes: depression and anxiety reduced; measures: DASS and ATQ^s^
Feinstein et al [26], 2012, United States, high quality	Quantitative, short-term prospective cohort study, and questionnaire	Setting: undergraduate university students; sample: 301 students (62% female; mean age 19.44, SD 2.05, years; 41% Asian or Pacific Islander, 41% White, 6% Latino, 6% African American, and 6% Other); presenting condition: some participants had raised depression, anxiety, or social anxiety at baseline	Social networking in general	Outcome: depression resulted in poor-quality social connections, which in turn worsened depression and anxiety; measures: DASS and BFNE^t^
Felnhofer et al [34], 2018, Austria, —	Quantitative, randomized controlled trial, and questionnaire	Setting: public university; sample: 95 students (87% female; mean age 23.34, SD 2.727, years; ethnicity unknown); presenting condition: none in particular	Avatars (virtual entities controlled by another human being) and agents (virtual entities controlled by a computer)	Outcome: social interaction anxiety unchanged; measure: SIAS^u^
Frison and Eggermont [37], 2016, Belgium, medium quality	Quantitative, uncontrolled cross-sectional, and questionnaire	Setting: 18 randomly selected high schools in Flanders, Belgium; sample: 910 students with Facebook account (52% female; average age 15.44, SD 1.71, years; ethnicity unknown; country of birth: 96.1% Belgium, 1.8% Europe, and 2.1% non-European country); presenting condition: none in particular	Facebook	Outcome: depression reduced; measure: CES-DC^v^
Garrido et al [43], 2019, Australia, medium quality	Qualitative and focus groups	Setting: high schools and universities in Western Australia; sample: 23 students (65% female; aged 13-25 years; ethnicity unknown); presenting condition: DASS score <15 (severely depressed excluded)	A total of 6 currently available smartphone apps for mental health (Mood Mission, Music eScape, Pacifica, Mindshift, Headspace, and What’s Up)	Outcome: helpful and unhelpful aspects of smartphone apps for mental health; measure: thematic analysis of focus group content
Horgan et al [54], 2013, Ireland, medium quality	Qualitative and quantitative, pre- and posttest and qualitative descriptive designs, extraction of posts from website, and questionnaire for CES-D scores	Setting: University of Cork; sample: 118 students (36% female; aged 18-24 years; 98.3% White and 1.7% Asian or Asian Irish); presenting condition: depression	Depression support website with peer support forum	Outcome: depression reduced; measure: CES-D
Horgan and Sweeney [18], 2010, Ireland, medium quality	Quantitative, descriptive study, and questionnaire	Setting: university; sample: 922 students (62% female; aged 18-24 years; ethnicity unknown); presenting condition: none in particular	Internet use for mental health support	Outcome: reasons for use of internet-based mental health support; measure: self-developed questionnaire
Lim et al [57], 2019, Australia, high quality	Qualitative and quantitative, descriptive design, pre- and posttest questionnaires, mood tracker, and semistructured interview	Setting: local youth health service (participants with social anxiety disorder) and Australian university (participants without social anxiety disorder); sample: 20 participants (45% female; aged 18-23 years; 91% White and 9% multiracial or other); presenting disorder: with or without social anxiety disorder	+Connect, a digital smartphone app with video material	Outcomes: depression and anxiety reduced; measures: CES-D and SIAS
Liu and Yu [35], 2013, Taiwan, medium quality	Quantitative, cross-sectional study, and questionnaire	Setting: college; sample: 330 Facebook-using students (63% female; aged 18-23 years; ethnicity unknown); presenting condition: none in particular	Facebook	Outcome: psychological well-being improved; measure: Ryff scales of psychological well-being
McCloskey et al [23], 2015, United States, medium quality	Quantitative, uncontrolled single-group design, and questionnaire	Setting: university; sample: 633 undergraduate students with Facebook page (70% female; aged ≥18 years, median age 21 years; 64.8% White); presenting condition: none in particular; participants on average had mild levels of depression at baseline	Facebook	Outcome: depression reduced; measure: PHQ-9
Mikami [38], 2010, United States, high quality	Quantitative, longitudinal, observation, and questionnaire	Setting: public middle school; sample: 92 social networking site users (58% female; mean age 20.92, SD 1.11, years; 58% White, 29% African American, and 13% Other or Mixed); presenting condition: none in particular	Web-based social networking	Outcome: depression—no outcomes reported; measure: CDI^w^
Ozcan and Buzlu [39], 2007, Turkey, high quality	Quantitative, uncontrolled single-group design, and questionnaire	Setting: university; sample: 730 undergraduate students who use the internet (53% female; mean age 20.84, SD 1.95, years; ethnicity unknown); presenting condition: none in particular	Internet use in general	Outcome: depression reduced; measure: BDI
Poppelaars [33], 2018, The Netherlands, medium quality	Quantitative, randomized controlled trial, and questionnaire	Setting: university; sample: 146 undergraduate students who play video games (71% female; mean age 20.2, SD 1.74, years; ethnicity unknown; nationality: 76% Dutch, 23% German, and 1% Other); presenting condition: none in particular; some with higher depressive symptoms at outset	Video game that included cooperation with other players and with mental health messaging vs video game without mental health messaging	Outcome: psychological well-being improved, with larger improvement for those higher in depressive symptoms; measures: BDI-II, SAM^x^, and International PANAS short form
Radovic [44], 2017, United States, United States, high quality	Qualitative, randomized controlled trial, semistructured interviews, think aloud, advisory boards, and focus groups	Setting: academic adolescent medicine clinic and specialty psychiatry clinic; sample: 23 patients (78% female; aged 13-20 years, mean age 16, SD 2.3, years); presenting condition: depression	Social media website for depressed adolescents	Outcome: adolescent-informed design of social media website for depression; measure: thematic analysis from semistructured interviews
Radovic [53], 2017, United States, medium quality	Qualitative, uncontrolled cross-sectional study, and semistructured interview	Setting: academic adolescent medicine clinic and specialty psychiatry clinic; sample: 23 patients (78% female; aged 13-20 years, mean age 16, SD 2.3, years; 87% White); presenting condition: depression	Social media	Outcomes: depressive symptoms either made participants reach for social media as a distraction or avoid it to avoid bringing down others. Psychological well-being improved; measure: thematic analysis from semistructured interviews
Rice et al [29], 2018, Australia, medium quality	Quantitative, uncontrolled single-group pilot, structured clinical interview, and questionnaire	Setting: mental health clinic; sample: 42 patients (50% female; aged 15-25 years, mean age 18.5, SD 2.1, years; ethnicity unknown; country of birth: 95.2% Australia); presenting condition: previous depression sufferers	Novel, moderated web-based social therapy intervention: Rebound	Outcomes: depression reduced and anxiety unchanged; measures: MADRS and DASS
Rice et al [31], 2020, Australia, high quality	Quantitative, single-group uncontrolled pre-post design, and questionnaire	Setting: 4 Headspace early intervention centers in northwestern Melbourne; sample: 89 patients (47% female; aged 14-25 years; ethnicity unknown); presenting condition: social anxiety	Social networking platform for socially anxious young people (Entourage): a *wall* function allows posting and commenting publicly	Outcomes: depression and social anxiety reduced and psychological well-being improved; measures: PHQ-9, MDRS-22^y^, LSAS^z^, BFNE, SIAS, and SWEMWBS^aa^
Santesteban-Echarri et al [46], 2017, Australia, medium quality	Qualitative, uncontrolled single-group pilot, semistructured interview, and focus group data	Setting: mental health clinic; sample: 42 patients (50% female; aged 15-25 years, mean age 18.5, SD 2.1, years; ethnicity unknown; country of birth: 95.2% Australia); presenting condition: previous depression sufferers	Novel, moderated web-based social therapy intervention: Rebound	Outcome: efficacy and usability evaluation of web-based social therapy intervention; measure: thematic analysis from semistructured interviews
Saulsberry et al [40], 2013, United States, medium quality	Quantitative, randomized controlled trial, and telephone interview	Setting: 12 primary care sites across southern and midwestern United States; sample: 58 patients (57% female; mean age 17.26, SD 1.85, years; 61% White, 24% Black, 6% Asian, 5% Hispanic, and 4% Other); presenting condition: depression	Primary care provider motivational interview+CATCH-IT internet program vs primary care provider brief advice+CATCH-IT internet program	Outcome: depression reduced; measures: CES-D-10, DSM-IV-TR^ab^, and PHQ-A^ac^
Selkie et al [47], 2020, United States, high quality	Qualitative, uncontrolled single-group design, and semistructured interviews	Setting: pediatric gender clinic; sample: 25 transgender adolescents with social media profile (44% trans-feminine; aged 15-18 years, mean age 16 years; 80% White non-Hispanic, 4% African American, 8% American Indian, and 8% Asian); presenting condition: none in particular	Social media platforms, including YouTube, Instagram, Facebook, Twitter, and Tumblr	Outcomes: positive and negative outcomes of using social media for mental health support; measure: —
Sharabi and Margalit [32], 2011, Israel, medium quality	Quantitative, cross-sectional crossover, and questionnaire	Setting: middle to high socioeconomic families vs those who failed in school (mostly from low socioeconomic families); sample: 716 students (48% female; aged 16-18 years; ethnicity unknown); presenting condition: with or without learning disabilities	Internet communication	Outcomes: psychological well-being negatively correlated with loneliness. Loneliness reduced by internet communication with people known offline; measure: Hebrew adaptation of Mood Scale
Sharabi and Margalit [19], 2011, Israel, medium quality	Quantitative and cross-sectional case-control	Setting: 3 high schools in urban Israel; sample: 887 students grades 10-12 (50% female; aged 16-18 years; ethnicity unknown); presenting condition: with (n=213) or without (n=674) learning disabilities	Internet communication	Outcome: psychological well-being reduced; measure: Hebrew adaptation of Affect Scale
Siriaraya et al [52], 2011, Cyprus, medium quality	Qualitative, cross-sectional study, and content analysis	Setting: general; sample: 400 messages from teenagers using web-based discussion forum (gender unknown; age range unknown; ethnicity unknown); presenting condition: none in particular	Web-based anonymous discussion forum	Outcome: level of support provided among adolescents; measure: Content analysis of web-based forum messages
Stockdale and Coyne [27], 2020, United States, high quality	Quantitative, longitudinal, and questionnaire	Setting: longitudinal study of intrafamily life participants; sample: 385 participants who use smartphones (53% female; aged 17-19 years; 70% European-American, 10% African American, 12% Multiethnic, 5% Asian American, and 2% Other); presenting condition: none in particular	Social media use	Outcomes: depression unchanged and anxiety worsened; measures: CES-DC and SCAS^ad^
van Rensburg et al [50], 2015, United States, high quality	Qualitative, uncontrolled single-group design, and semistructured interviews	Setting: Yale Psychiatric Hospital Intensive Outpatient Program; sample: 20 patients (75% female; aged 14-19 years; 80% White, 15% Hispanic, and 5% Mixed); presenting condition: combination of ADHD^ae^, mood disorder NOS^af^, MDD^ag^, anxiety, PTSD^ah^, psychosis, and ODD^ai^	Social media for patient-provider interactions	Outcomes: positive (including safety) and negative (including anxiety) outcomes of patient-provider interactions through social media; measure: thematic analysis of semistructured interviews
van Zalk et al [41], 2011, Sweden, high quality	Quantitative, uncontrolled single-arm longitudinal study, and questionnaire	Setting: university in Utrecht; sample: 197 psychology freshmen (78% female; mean age 18.9, SD 1.6, years; ethnicity unknown; 92% Dutch origin); presenting condition: none in particular	Web-based chatting with friends through web-based social networking site	Outcome: depression unchanged; measure: BDI Dutch short version
Van Zalk and Tillfors [21], 2017, Sweden, high quality	Quantitative, longitudinal study, and questionnaire	Setting: Swedish school; sample: 526 students from grades 7-9 (68% female; aged 13-15 years; ethnicity unknown; 12.1% first-generation immigrants); presenting condition: none in particular	Web-based chatting with friends through web-based social networking site	Outcome: Reduced depression among those with higher, but not lower, social anxiety; measures: CES-D and SPSQ-C^aj^
Wright et al [42], 2013, United States, medium quality	Quantitative, cross-sectional observational study, and questionnaire	Setting: undergraduate university; sample: 361 students who use Facebook (53% female; mean age 20.26, SD 2.72, years; 77% White, 8.6% Native American, 4.4% Latino, 3.6% Asian American, 3.3% African American, and 3.3% Other); presenting condition: none in particular	Facebook use	Outcome: depression reduced; measure: CES-D
Yeh et al [20], 2008, Taiwan, medium quality	Quantitative, cross-sectional, and questionnaire	Setting: project of mental health survey; sample: 3477 college students (55% female; mean age 22.45, SD 1.56, years; ethnicity unknown); presenting condition: none in particular	Social support on the web	Outcome: depression worsened by higher web-based and lower actual social support; measure: Ko Depression Inventory

^a^BPRS: Brief Psychiatric Rating Scale.

^b^CDRS: Children’s Depression Rating Scale.

^c^BAI: Beck Anxiety Inventory.

^d^PACE: Personal Assessment and Crisis Evaluation.

^e^SWLS: Satisfaction With Life Scale.

^f^MADRS: Montgomery–Åsberg Depression Rating Scale.

^g^PSSS: Perceived Social Support Scale.

^h^PHQ-9: Patient Health Questionnaire Depression Scale.

^i^HADS: Hospital Anxiety and Depression Scale.

^j^CES-D: Center for Epidemiological Studies Depression Scale.

^k^RCMAS: Revised Children’s Manifest Anxiety Scale.

^l^MPPS: Mothers’ Perceptions of Mobile Phone–Based Peer Support.

^m^SASC-R: Social Anxiety Scale for Children-Revised.

^n^Not available.

^o^DASS: Depression Anxiety Stress Scales.

^p^CTI: Cognitive Triad Inventory.

^q^BDI-II: Beck Depression Inventory II.

^r^PANAS: Positive and Negative Affect Scale.

^s^ATQ: Automatic Thoughts Questionnaire.

^t^BFNE: Brief Fear of Negative Evaluation.

^u^SIAS: Social Interaction Anxiety Scale.

^v^CES-DC: Center for Epidemiological Studies Depression Scale for Children.

^w^CDI: Children’s Depression Inventory.

^x^SAM: Self-Assessment Manikin.

^y^MDRS-22: Male Depression Risk Scale.

^z^LSAS: Liebowitz Social Anxiety Scale.

^aa^SWEMWBS: Short Warwick–Edinburgh Mental Well-being Scale.

^ab^DSM-IV-TR: Diagnostic and Statistical Manual of Mental Disorders, 4th Edition, Text Revision.

^ac^PHQ-A: Patient Health Questionnaire-9 modified for Adolescents.

^ad^SCAS: Spence Children’s Anxiety Scale.

^ae^ADHD: attention-deficit/hyperactivity disorder.

^af^NOS: not otherwise specified.

^ag^MDD: major depressive disorder.

^ah^PTSD: posttraumatic stress disorder.

^ai^ODD: oppositional defiant disorder.

^aj^SPSQ-C: Social Phobia Screening Questionnaire for Children and Adolescents.

### Indicators That QSC Is Present in Digital Interventions

Indicators and measures used to quantitatively assess D-QSC presence were heterogeneous (Tables 3 and 4). The most common indicator for D-QSC was social support (14/42, 33%; Table 3). Among the 31 quantitative studies assessed, there were 20 different standardized questionnaires used to measure QSC (Tables 3 and 4), with only 4 (13%) studies using the same measure (Multidimensional Scale of Perceived Social Support). Nonstandardized questionnaires were also used in some studies, including single questions (eg, “I hope to gain support through meeting people going through similar experiences, Y/N”). The remaining indicators of QSC were identified from qualitative analysis within 17 studies [43-59].

**Table 3:** Indicators of the presence of quality social connection within digital interventions in the included studies (N=42).

Indicator	Description and measurement example	Values, n (%)	Improved depression outcomes, n (%)	Improved anxiety outcomes, n (%)
Social support^a^	Level of support received from others. Multidimensional Scale of Perceived Social Support: “There is a special person who is around when I am in need”	14 (33) [20,23,24,30,35-39,41,42,46,53,56]	9 (64) [23,24,30,36,37,39,41,42,56]	2 (14) [24,56]
Social connectedness^a^	A sense of feeling connected to others. Social Connectedness Scale Revised: “I feel understood by the people I know”	10 (24) [22,27-29,31,43,52,56-58]	6 (60) [22,28,29,31,57,58]	5 (50) [28,31,52,57,58]
Relatedness	Bonding through shared experience or understanding. Open-ended survey questions to determine best and worst aspects of intervention	5 (12) [33,43,46,55,56]	1 (20) [56]	1 (20) [56]
Connecting with similar people	Communicating with those who have similar experiences and feelings. Content analysis and thematic coding of qualitative questions	4 (10) [18,25,47,53]	1 (25) [25]	1 (25) [25]
Feeling accepted	Having a sense that people are okay with, and accepting of, oneself. Likert-scale response to statement “I felt that the [forum] moderators accepted me”	3 (7) [29,53,59]	2 (67) [29,59]	1 (33) [59]
Being able to share	Feeling able to disclose one’s thoughts and feelings to others. Friendship Quality Questionnaire: “I would tell him or her what upsets me”	4 (10) [21,51,52,54]	2 (50) [21,54]	1 (25) [52]
Feeling normalized	Someone making it clear that what one is feeling is normal. Peer Support Evaluation Inventory subscale item: “Helped me feel that what I was going through was ‘normal’”	3 (7) [47,52,59]	1 (33) [59]	2 (67) [52,59]
Feeling close to a peer	A sense of intimacy or connection with another person. Peer Support Evaluation Inventory subscale item: “I felt close to my peer”	2 (5) [57,59]	2 (100) [57,59]	2 (100) [57,59]
Less alone in one’s feelings	Knowing that others are experiencing similar feelings. Content analysis and thematic coding of qualitative interview questions	3 (7) [25,54,55]	2 (67) [25,54]	1 (33) [25]
Sense of belonging	Feeling that one is part of a group. Interpersonal Needs Questionnaire: “I don’t fit in”	2 (5) [22,31]	2 (100) [22,31]	1 (50) [31]
Emotional connection	A bond created among 2 or more people by sharing feelings. Text-based ethnographic study of instant messaging conversations	2 (5) [48,50]	—^b^	—
Empathy	Understanding and sharing feelings of another person. Networked Minds Measure of Social Presence Empathy subscale: “When the other was happy, I was happy”	2 (5) [27,34]	—	—
Feeling you are not a burden	Sense that one is not bothering or troubling others. Interpersonal Needs Questionnaire low score for items such as “These days I think I make things worse for the people in my life”	2 (5) [22,31]	2 (100) [22,31]	1 (50) [31]
Rapport	Trust and understanding established between the provider and patient. Provider-reported from ethnography	1 (2) [45]	—	—
Feeling validated	Having acceptance or approval from others of one’s thoughts and feelings. Content analysis and thematic coding of qualitative questions, categorized as *Appraisal support*	1 (2) [47]	—	—
Shared understanding	Another person knowing how one is feeling through their own similar experience. Content analysis and thematic coding of forum posts	1 (2) [54]	1 (100) [54]	—
Trust	Ability to rely on someone. Peer Support Evaluation Inventory: “My peer was trustworthy”	1 (2) [59]	1 (100) [59]	1 (100) [59]

^a^Directly encapsulates the definition of quality social connection.

^b^Not available.

**Table 4:** Indicators of the absence of quality social connection within digital interventions in the included studies (N=42).

Indicator	Description and measurement example	Values, n (%)	Improved depression outcomes, n (%)	Improved anxiety outcomes, n (%)
Negative interactions	Harm being inflicted through digital interventions, resulting in negative feelings such as loneliness or hurt. Social Networking Survey: “How positive (or negative) are your interactions with people on FB^a^ and MS^b^?”	6 (14) [26,43,47,49,51,53]	—^c^	—
Loneliness	A sense of isolation as a result of being disconnected from other people. University of California, Los Angeles, Loneliness Scale: “I lack companionship”	7 (17) [19,30-32,40,47,59]	4 (57) [30,31,40,59] (Reduced loneliness)	2 (29) [31,59] (Reduced loneliness)
Feeling ignored	Not being responded to. Content analysis and thematic coding of semistructured interviews exploring engagement with therapist through social networks and its efficacy	2 (5) [49,51]	—	—

^a^FB: Facebook.

^b^MS: Myspace.

^c^Not available.

### Associations Between D-QSC and Outcomes

**Figure 2:**
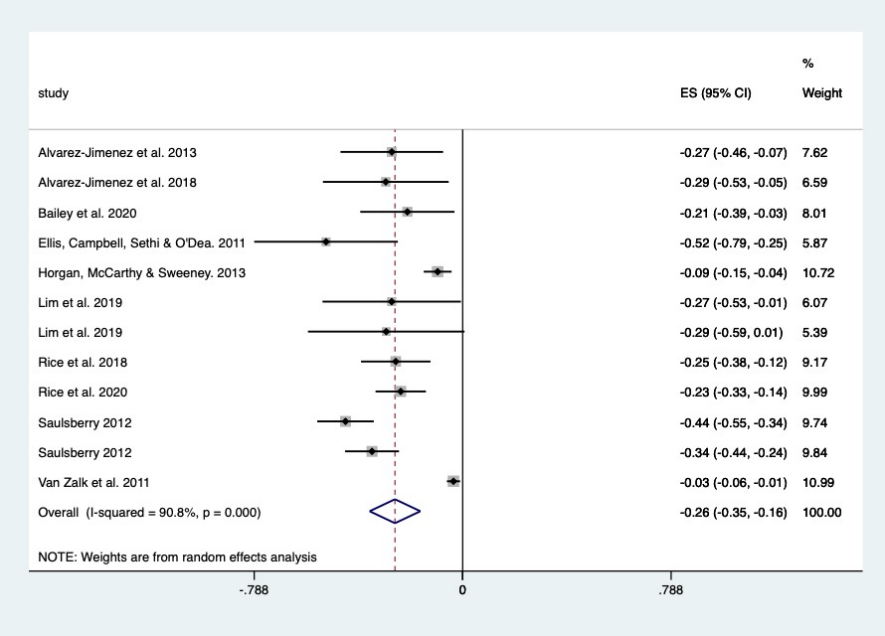
Forest plot showing the effect of social connection within digital interventions on depression outcomes. ES: effect size.

**Figure 3:**
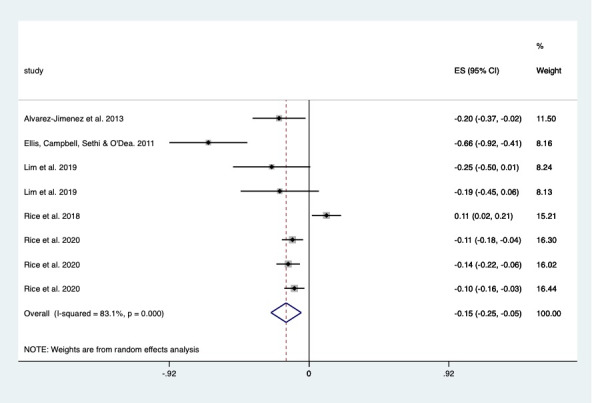
Forest plot showing the effect of social connection within digital interventions on anxiety outcomes. ES: effect size.

The relationship between D-QSC indicators and outcomes was mixed. Of the 42 studies, 10 (24%) reported a change in depression symptoms over time after participants experienced a D-QSC, and a pooled analysis demonstrated a significant weighted mean decrease in depression by 25.6% (–0.256, 95% CI –0.352 to –0.160; *P*<.001), with high heterogeneity (I^2^= 90.8%; Figure 2) [20,23,24,26,30,36,37,39,41,42]. Of the 42 studies, 5 (12%) reported change over time in anxiety symptoms; there was also a decrease, but it was smaller (15%; –0.151, 95% CI –0.251 to –0.051; *P*=.003), with high heterogeneity (I^2^= 83.1%; Figure 3) [29,31,56-58].

The indicators of D-QSC associated with improved depression or anxiety symptoms included social support [23,24,30,36,37,39,41,42,56], social connectedness [22,28,29,31,52,57,58], loneliness (reduced) [30,31,40,54,59], relatedness [56], sense of belonging [22,31], being able to share [21,52,54], less alone in one’s feelings [25,54], feeling normalized [52,59], feeling close to peer [57,59], feeling you are not a burden [22,31], feeling accepted [29,59], shared understanding [54], and trust [59] (Table 3). For example, depression outcomes improved after good social support for those abused on the web [36] and for adolescents with high social anxiety [21]. In contrast, negative interactions [20,26], negative experiences of social support [49,51,52], and frequent social media use [27] were associated with worsened outcomes (Table 4). For example, young people aged 17-19 years using social media (eg, Facebook, Instagram, and Twitter) to connect with others were more likely to have anxiety but not depression at 3 years’ follow-up [27]. A similar but older study found a worsening of depression outcomes with social networking interactions (ie, Facebook, Myspace, and texting) [26]. This relationship between negative interactions on social media and worsened outcomes was evident in both men and women, particularly in those also receiving low face-to-face social support [20]. Additional indicators of D-QSC that did not explicitly indicate effect on depression or anxiety were feeling validated [47], rapport [45], empathy [27,34], and emotional connection [48,50]. The indicators also improved well-being outcomes (Multimedia Appendix 4  [19,30-33,35,48,49,53]).

### Digital Intervention Mechanisms That Facilitate QSC

Digital intervention mechanisms mainly helped facilitate QSC [18,20,25,30,35,43,44,46,50,51,55,56,58,59]. Forum moderation, confidentiality, ease of access, and anonymity supported by digital interventions were cited as valuable [18,43,50,56,58,59], facilitated open sharing in digital environments, and could lower inhibitions compared with face-to-face engagement [50,51]. The usual signals received during face-to-face interactions, such as body language or facial expression, were lost during digital interactions [52] and could impair the quality of interactions [26].

D-QSC was deemed more valuable when digital interactions were blended with face-to-face interactions [20,35,43]. For example, digital interactions were convenient and accessible, whereas face-to-face meetings helped maintain the connection. Higher levels of web-based social support were associated with increased symptoms of depression, specifically in both men and women and those who had little in-person social support [20]. One study found that participants were disconnected from the *real world* through high levels of web-based engagement [43]. Indeed, disconnection can have an impact on the interaction between peers and family and result in increased loneliness [19,32]. Other studies indicated that participants felt ignored [51], misunderstood [51], and had hurt feelings [43,49].

Participants also valued opportunities to support others [41,55], to connect with peers, and compare similar mental health experiences [25,54]. Some participants considered the networking component as the most helpful aspect of a moderated web-based social therapy tool, more helpful than the therapy itself [46]. Harassment was also identified as occurring frequently on the web. For example, a study reported this frequently among transgender adolescents [32].

### Individual and Contextual Factors Influencing Mechanisms

#### Demographic and Personality Factors

The effect of D-QSC on depression outcomes differed across genders and personality variables. Social support from active Facebook use predicted a reduction in depression symptoms in girls but not in boys [37]. In another study, increased social support on the web and decreased offline social support was associated with increased depression symptoms across both genders [20]. Demographic (eg, personality type) and dynamic (eg, vulnerability level) characteristics were also reported to modulate the influence of D-QSC on depression and anxiety outcomes. Personality differences were only discussed in 5% (2/42) of the studies [41,48]. Chatting exclusively on the web predicted significantly improved depression [41] or psychological well-being [48] outcomes only in participants with more introverted personality traits.

#### Anxiety Versus Depression

D-QSC was more important for depression than for anxiety outcomes. For example, both web-based self-help cognitive behavioral therapy and peer support effectively reduced anxiety, but peer support was more effective in improving outcomes of depression [56]. Moreover, those with higher social anxiety had lower depression symptoms after corumination with a web-based best friend [21]. In contrast, symptoms of depression predicted negative social networking interactions, which in turn resulted in higher symptoms of depression and anxiety [26]. An app designed to strengthen relationships and increase social connections for individuals with social anxiety disorder also improved symptoms of depression [57]. This effect lasted longer in participants without existing social anxiety disorder.

#### Offline–Web-Based Engagement

A cross-sectional study reported improved mood only for participants chatting with friends on the web who were also known offline; they were not web-exclusive friends [32]. Social web-based gamers who had lower depression and social anxiety on the web had higher QSC with friends offline [28].

### Adapted Conceptual Framework

#### Stage 1

A total of 55 indicators were found from professionals (19/55, 35%), young people (19/55, 35%), and the literature (17/55, 30%; Multimedia Appendix 5). Social connectedness and social support were excluded because they directly described social connection and were not *attributes* of D-QSC (indicators). Of the 55 indicators, 5 (9%) were direct repeats and 5 (9%) were deemed not attributes of D-QSC.

#### Stage 2

The remaining 45 indicators were grouped if conceptually similar or stood alone. For example, *Trust established* and *Trust*, as well as *Nonjudgmental* and *Not feeling judged,* were grouped, respectively. After the grouping, 30 indicators remained.

#### Stage 3

**Figure 4:**
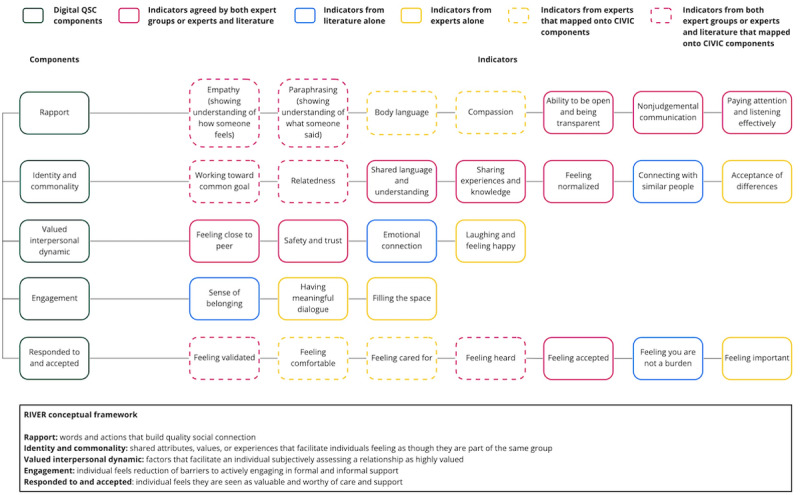
Adapted RIVER (rapport, identity and commonality, valued interpersonal dynamic, engagement, and responded to and accepted) conceptual framework for quality social connection within digital interventions. CIVIC: Closeness, Identity and common bond, Valued relationships, Involvement, and Cared for and accepted; QSC: quality social connection.

Of the 30 D-QSC indicators, 10 (33%) were initially mapped directly onto the preexisting CIVIC framework components (Figure 4). The remaining 67% (20/30) of indicators that did not map on directly were either merged with indicators that naturally went together, such as *Safety* and *trust*, or remained standalone indicators. Merged indicators (eg, *Safety and trust*, *Feeling close to peer*, and *Laughing and feeling happy*) and standalone indicators (eg, *Emotional connection*) were then loosely grouped and given new provisional component names (eg, Valued interpersonal dynamic) that suited the indicators’ collective meaning.

#### Stage 4

Young experts reviewed the preliminary framework and identified 2 extra indicators that were deemed important (ie, *Feeling important* and *Acceptance of differences*) and added to the framework. Experts also helped to refine the wording or further merge indicators and components. For example, the *Identity and common bond* component was changed to *Identity and commonality*. There were 28 indicators across 5 components: rapport, identity and commonality, valued interpersonal dynamic, engagement, and responded to and accepted, given the acronym RIVER (Figure 4).

## Discussion

### Principal Findings

To our knowledge, this is the first systematic scoping review with meta-analysis to examine D-QSC as an *active ingredient* for depression and anxiety outcomes in young people. Usually conflated with quantity of social connections, QSC has now been comprehensively examined for its relevance to the mental health outcomes of digital interventions. We coproduced a conceptual framework of D-QSC for young people experiencing depression or anxiety that summarizes current understanding of component attributes or indicators. The RIVER framework comprises indicators relevant for establishing and assessing the presence of D-QSC. This can be characterized across 5 components. These components are interconnected and may not be exhaustive, but they provide a foundation for further work in this field to establish appropriate metrics for D-QSC.

D-QSC seems to help improve depression outcomes across most digital interventions. However, there is weaker evidence that D-QSC improves anxiety and well-being. There was also limited evidence of gaming, which was surprising considering that the participative nature with other users is at its core. D-QSC also worsened depression and anxiety outcomes in some instances, but this was often a result of negative interactions through social networking sites, which could be construed as a poor D-QSC. Few studies examined individual factors, contextual factors, or digital mechanisms that may modulate the impact of digital QSC on mental health outcomes. However, in the few studies that did report on mechanisms, a face-to-face connection before web-based support was an important consideration for improving outcomes. Furthermore, the impact of web-based support was modulated by the strength of offline connections.

### Comparison With Prior Work

Reviews assessing the efficacy of digital mental health interventions for young people have found digital interventions to be as efficacious as, or sometimes more efficacious than, similar interventions delivered in person [65]. The strongest review to date that most closely relates to QSC collated measures of *social connectedness* to produce a conceptual framework of social connectedness in mental disorders (CIVIC) [17]. However, the review positioned social connectedness as the solution to loneliness and not as an *active ingredient* for the prevention and treatment of mental disorders (ie, depression and anxiety). Our work extended this framework to ensure that QSC was considered for digital interventions, for young people, and across different contexts. This process substantially expanded the elements indicating the development or presence of D-QSC and required redefining the CIVIC framework components to form the *RIVER framework of D-QSC indicators for young people*. Interestingly, the component of the CIVIC framework found to be most frequently assessed in current QSC metrics was *Identity and common bond*; this was the only component of our adapted framework not selected as a top priority in the context of digital interventions for anxiety and depression during review of the framework by 9 young people. This highlighted the need to develop improved metrics that are uniformly applied.

### Strengths and Limitations

Young people with lived experience were involved at all review stages, including screening and interpretation. Dual independent review of the literature with people with direct experience of the review area helped us to overcome some limitations inherent in the current literature to gain better understanding of QSC and ensure accuracy of the screening. The insight from the young people during the data synthesis and interpretation stages helped to retain the data integrity. We have subsequently added to the limited evidence base for the impact of patient and public involvement throughout all stages of systematic and scoping reviews. Our adapted RIVER framework provides the foundation for future work to develop measures that would enable a developer, evaluator, or practitioner using digital interventions for mental health to assess the presence and degree of the QSC established.

The main limitation was that the studies did not control for a previous established connection offline before the D-QSC. Of the 42 studies, only 12 (29%) could be included in the meta-analysis because of a lack of measured effect sizes in previous work and heterogeneity across approaches, suggesting that the results should be interpreted with caution. Because of the scoping nature of the review, there was also statistical and methodological variability in the meta-analysis. Only manuscripts that were in English were included, which enables cultural bias. However, this was mitigated to an extent by working with young people and professionals from a variety of countries and cultural contexts to interpret the findings.

### Clinical and Research Implications

QSC should be considered in the development and application of most digital interventions, particularly for depression. However, more research is needed to examine its impact within gaming platforms. In general, digital interventions mostly helped facilitate QSC; therefore, developers should consider factors such as user preference, anonymity, delivery medium, and content moderation. Initially, they should consider whether D-QSC is appropriate, depending on the target audience, and whether it will be important for engagement, or efficacy, or both. Further research is required to establish which individuals, conditions, and therapeutic mechanisms respond most strongly to D-QSC and what format is most appropriate. Clinical trials of any new digital intervention for mental health should control for previous face-to-face connections.

Future research should build upon our RIVER framework to further examine relationships among individual indicators of QSC, variations across different digital interventions, and the impact on outcomes across different user groups, particularly those in low- and middle-income countries. Factors that may mediate any causative relationships between QSC and mental health outcomes also deserve further attention. This work will inform the creation of standardized measures for D-QSC to evaluate its presence across different social settings. New measures should be developed to assess (1) attributes of a digital intervention that help or hinder good QSC and (2) the perceived value of a particular QSC for an individual and its relationship to outcomes within digital interventions. This work has value for development, regulation, and evaluation of digital mental health interventions, as well as delineating helpful and harmful web-based interactions for young people, including social media. It will be vital to expand digital mental health care provision during the COVID-19 pandemic.

As the COVID-19 pandemic accelerates the shift to digital delivery of traditional therapy [13], clinicians should be trained in how to incorporate techniques for developing or maintaining D-QSC. Guidelines should be developed to ensure that moving face-to-face therapies to web-based spaces does not affect the QSC in the practitioner-patient dyad, and they should include strategies to improve connection on the web. Further clinical recommendations include a prioritization of video communication for web-based therapy to allow body language to be observed. However, anonymity can be beneficial to some users when first divulging sensitive mental health information. Blended care should enable patients to first meet their therapist in person, if desired, to facilitate QSC that can be translated to digital follow-ups.

### Conclusions

In conclusion, D-QSC is important and an underconsidered component supporting engagement and efficacy for young people with depression and anxiety. In the wake of the COVID-19 pandemic, our work holds relevance as mental health needs rise and support will increasingly be provided on the web.

## Appendix

Multimedia Appendix 1 PRISMA (Preferred Reporting Items for Systematic Reviews and Meta-Analyses) checklist.

Multimedia Appendix 2 Stepwise approach to developing a full and objective search strategy.

Multimedia Appendix 3 Expanded data extraction and data quality assessment.

Multimedia Appendix 4 Supplementary analyses.

Multimedia Appendix 5 Indicators of social connection within digital interventions that contributed to the conceptual framework.

## Abbreviations

CIVIC: closeness, identity and common bond, valued relationships, involvement, and cared for and accepted
D-QSC: quality social connection within digital interventions
PRISMA: Preferred Reporting Items for Systematic Reviews and Meta-Analyses
QSC: quality social connection
RIVER: rapport, identity and commonality, valued interpersonal dynamic, engagement, and responded to and accepted
YPAG: Young Persons Advisory Group

## References

[1] Kessler RC, Berglund P, Demler O, Jin R, Merikangas KR, Walters EE (2005). Lifetime prevalence and age-of-onset distributions of DSM-IV disorders in the National Comorbidity Survey Replication. Arch Gen Psychiatry.

[2] Vigo D, Thornicroft G, Atun R (2016). Estimating the true global burden of mental illness. Lancet Psychiatry.

[3] Pfefferbaum B, North CS (2020). Mental health and the Covid-19 pandemic. N Engl J Med.

[4] Pierce M, Hope H, Ford T, Hatch S, Hotopf M, John A, Kontopantelis E, Webb R, Wessely S, McManus S, Abel KM (2020). Mental health before and during the COVID-19 pandemic: a longitudinal probability sample survey of the UK population. Lancet Psychiatry.

[5] Choi KW, Stein MB, Nishimi KM, Ge T, Coleman JR, Chen C, Ratanatharathorn A, Zheutlin AB, Dunn EC, Breen G, Koenen KC, Smoller JW, 23andMe Research Team, Major Depressive Disorder Working Group of the Psychiatric Genomics Consortium (2020). An exposure-wide and mendelian randomization approach to identifying modifiable factors for the prevention of depression. Am J Psychiatry.

[6] Cruwys T, Alexander Haslam S, Dingle GA, Jetten J, Hornsey MJ, Desdemona Chong E, Oei TP (2014). Feeling connected again: interventions that increase social identification reduce depression symptoms in community and clinical settings. J Affect Disord.

[7] (2020). Therapeutic alliance—APA dictionary of psychology. American Psychology Association.

[8] Murphy R, Hutton P (2018). Practitioner review: therapist variability, patient-reported therapeutic alliance, and clinical outcomes in adolescents undergoing mental health treatment - a systematic review and meta-analysis. J Child Psychol Psychiatry.

[9] Pihlaja S, Stenberg J, Joutsenniemi K, Mehik H, Ritola V, Joffe G (2018). Therapeutic alliance in guided internet therapy programs for depression and anxiety disorders - a systematic review. Internet Interv.

[10] Kellezi B, Wakefield JR, Stevenson C, McNamara N, Mair E, Bowe M, Wilson I, Halder MM (2019). The social cure of social prescribing: a mixed-methods study on the benefits of social connectedness on quality and effectiveness of care provision. BMJ Open.

[11] Levula A, Wilson A, Harré M (2016). The association between social network factors and mental health at different life stages. Qual Life Res.

[12] Holt-Lunstad J, Smith TB, Layton JB (2010). Social relationships and mortality risk: a meta-analytic review. PLoS Med.

[13] Chang BP, Kessler RC, Pincus HA, Nock MK (2020). Digital approaches for mental health in the age of covid-19. BMJ.

[14] Hollis C, Morriss R, Martin J, Amani S, Cotton R, Denis M, Lewis S (2015). Technological innovations in mental healthcare: harnessing the digital revolution. Br J Psychiatry.

[15] Sanger E (2020). Social networking in mental health interventions for adolescents. Perspect Public Health.

[16] Garett R, Lord LR, Young SD (2016). Associations between social media and cyberbullying: a review of the literature. Mhealth.

[17] Hare-Duke L, Dening T, de Oliveira D, Milner K, Slade M (2019). Conceptual framework for social connectedness in mental disorders: systematic review and narrative synthesis. J Affect Disord.

[18] Horgan A, Sweeney J (2010). Young students' use of the internet for mental health information and support. J Psychiatr Ment Health Nurs.

[19] Sharabi A, Margalit M (2011). Virtual friendships and social distress among adolescents with and without learning disabilities: the subtyping approach. Eur J Spec Needs Educ.

[20] Yeh Y-C, Ko H-C, Wu JY, Cheng C-P (2008). Gender differences in relationships of actual and virtual social support to internet addiction mediated through depressive symptoms among college students in Taiwan. Cyberpsychol Behav.

[21] Van Zalk N, Tillfors M (2017). Co-rumination buffers the link between social anxiety and depressive symptoms in early adolescence. Child Adolesc Psychiatry Ment Health.

[22] Bailey E, Alvarez-Jimenez M, Robinson J, D'Alfonso S, Nedeljkovic M, Davey CG, Bendall S, Gilbertson T, Phillips J, Bloom L, Nicholls L, Garland N, Cagliarini D, Phelan M, McKechnie B, Mitchell J, Cooke M, Rice SM (2020). An enhanced social networking intervention for young people with active suicidal ideation: safety, feasibility and acceptability outcomes. Int J Environ Res Public Health.

[23] McCloskey W, Iwanicki S, Lauterbach D, Giammittorio DM, Maxwell K (2015). Are Facebook "friends" helpful? Development of a Facebook-based measure of social support and examination of relationships among depression, quality of life, and social support. Cyberpsychol Behav Soc Netw.

[24] Blackwell L, Romero S, Romero C, McLean K, Dawkins K, Hoag J (2012). CFfone: a social networking site for adolescents and young adults with cf. Proceedings of the Pediatric Pulmonology - The 26th Annual North American Cystic Fibrosis Conference.

[25] Canady VA (2018). Survey explores social media, mental well-being among youth. Ment Health Wkly.

[26] Feinstein BA, Bhatia V, Hershenberg R, Davila J (2012). Another venue for problematic interpersonal behavior: the effects of depressive and anxious symptoms on social networking experiences. J Soc Clin Psychol.

[27] Stockdale LA, Coyne SM (2020). Bored and online: reasons for using social media, problematic social networking site use, and behavioral outcomes across the transition from adolescence to emerging adulthood. J Adolesc.

[28] Colder Carras M, Van Rooij AJ, Van de Mheen D, Musci R, Xue Q-L, Mendelson T (2017). Video gaming in a hyperconnected world: a cross-sectional study of heavy gaming, problematic gaming symptoms, and online socializing in adolescents. Comput Human Behav.

[29] Rice S, Gleeson J, Davey C, Hetrick S, Parker A, Lederman R, Wadley G, Murray G, Herrman H, Chambers R, Russon P, Miles C, D'Alfonso S, Thurley M, Chinnery G, Gilbertson T, Eleftheriadis D, Barlow E, Cagliarini D, Toh J, McAlpine S, Koval P, Bendall S, Jansen JE, Hamilton M, McGorry P, Alvarez-Jimenez M (2018). Moderated online social therapy for depression relapse prevention in young people: pilot study of a 'next generation' online intervention. Early Interv Psychiatry.

[30] Alvarez-Jimenez M, Gleeson J, Bendall S, Penn D, Yung A, Ryan R, Eleftheriadis D, D'Alfonso S, Rice S, Miles C, Russon P, Lederman R, Chambers R, Gonzalez-Blanch C, Lim M, Killackey E, McGorry P, Nelson B (2018). Enhancing social functioning in young people at Ultra High Risk (UHR) for psychosis: a pilot study of a novel strengths and mindfulness-based online social therapy. Schizophr Res.

[31] Rice S, O'Bree B, Wilson M, McEnery C, Lim MH, Hamilton M, Gleeson J, Bendall S, D'Alfonso S, Russon P, Valentine L, Cagliarini D, Howell S, Miles C, Pearson M, Nicholls L, Garland N, Mullen E, McGorry PD, Alvarez-Jimenez M (2020). Leveraging the social network for treatment of social anxiety: pilot study of a youth-specific digital intervention with a focus on engagement of young men. Internet Interv.

[32] Sharabi A, Margalit M (2011). The mediating role of internet connection, virtual friends, and mood in predicting loneliness among students with and without learning disabilities in different educational environments. J Learn Disabil.

[33] Poppelaars M, Lichtwarck-Aschoff A, Kleinjan M, Granic I (2018). The impact of explicit mental health messages in video games on players’ motivation and affect. Comput Hum Behav.

[34] Felnhofer A, Kafka JX, Hlavacs H, Beutl L, Kryspin-Exner I, Kothgassner OD (2018). Meeting others virtually in a day-to-day setting: investigating social avoidance and prosocial behavior towards avatars and agents. Comput Hum Behav.

[35] Liu C-Y, Yu C-P (2013). Can Facebook use induce well-being?. Cyberpsychol Behav Soc Netw.

[36] Cole DA, Nick EA, Zelkowitz RL, Roeder KM, Spinelli T (2017). Online social support for young people: does it recapitulate in-person social support; can it help?. Comput Human Behav.

[37] Frison E, Eggermont S (2015). Exploring the relationships between different types of facebook use, perceived online social support, and adolescents’ depressed mood. Soc Sci Comput Rev.

[38] Mikami AY, Szwedo DE, Allen JP, Evans MA, Hare AL (2010). Adolescent peer relationships and behavior problems predict young adults' communication on social networking websites. Dev Psychol.

[39] Ozcan NK, Buzlu S (2007). Internet use and its relation with the psychosocial situation for a sample of university students. Cyberpsychol Behav.

[40] Saulsberry A, Marko-Holguin M, Blomeke K, Hinkle C, Fogel J, Gladstone T, Bell C, Reinecke M, Corden M, Van Voorhees BW (2013). Randomized clinical trial of a primary care internet-based intervention to prevent adolescent depression: one-year outcomes. J Can Acad Child Adolesc Psychiatry.

[41] Van Zalk MH, Branje SJ, Denissen J, Van Aken MA, Meeus WH (2011). Who benefits from chatting, and why? The roles of extraversion and supportiveness in online chatting and emotional adjustment. Pers Soc Psychol Bull.

[42] Wright KB, Rosenberg J, Egbert N, Ploeger NA, Bernard DR, King S (2013). Communication competence, social support, and depression among college students: a model of facebook and face-to-face support network influence. J Health Commun.

[43] Garrido S, Cheers D, Boydell K, Nguyen QV, Schubert E, Dunne L, Meade T (2019). Young people's response to six smartphone apps for anxiety and depression: focus group study. JMIR Ment Health.

[44] Radovic A, DeMand AL, Gmelin T, Stein BD, Miller E (2018). SOVA: design of a stakeholder informed social media website for depressed adolescents and their parents. J Technol Hum Serv.

[45] Clarke CS (2018). Telepsychiatry in Asperger's syndrome. Ir J Psychol Med.

[46] Santesteban-Echarri O, Rice S, Wadley G, Lederman R, D'Alfonso S, Russon P, Chambers R, Miles CJ, Gilbertson T, Gleeson JF, McGorry PD, Álvarez-Jiménez M (2017). A next-generation social media-based relapse prevention intervention for youth depression: qualitative data on user experience outcomes for social networking, safety, and clinical benefit. Internet Interv.

[47] Selkie E, Adkins V, Masters E, Bajpai A, Shumer D (2020). Transgender adolescents' uses of social media for social support. J Adolesc Health.

[48] Dolev-Cohen M, Barak A (2013). Adolescents’ use of instant messaging as a means of emotional relief. Comput Human Behav.

[49] Bhuvaneswar CG, Gutheil TG (2008). E-mail and psychiatry: some psychotherapeutic and psychoanalytic perspectives. Am J Psychother.

[50] van Rensburg SH, Klingensmith K, McLaughlin P, Qayyum Z, van Schalkwyk GI (2016). Patient-provider communication over social media: perspectives of adolescents with psychiatric illness. Health Expect.

[51] Dhesi M (2019). A qualitative study to investigate in what ways are the distinctive features of synchronous text- based counselling experienced as being helpful and/or unhelpful by young people?. University of Roehampton.

[52] Siriaraya P, Tang C, Ang CS, Pfeil U, Zaphiris P (2011). A comparison of empathic communication pattern for teenagers and older people in online support communities. Behav Inf Technol.

[53] Radovic A, Gmelin T, Stein BD, Miller E (2017). Depressed adolescents' positive and negative use of social media. J Adolesc.

[54] Horgan A, McCarthy G, Sweeney J (2013). An evaluation of an online peer support forum for university students with depressive symptoms. Arch Psychiatr Nurs.

[55] Campbell A, Ridout B, Amon K, Navarro P, Collyer B, Dalgleish J (2019). A customized social network platform (Kids Helpline Circles) for delivering group counseling to young people experiencing family discord that impacts their well-being: exploratory study. J Med Internet Res.

[56] Ellis L, Campbell A, Sethi S, O'Dea B (2011). Comparative randomized trial of an online cognitive-behavioral therapy program and an online support group for depression and anxiety. J Cyber Ther Rehabil.

[57] Lim MH, Rodebaugh TL, Eres R, Long KM, Penn DL, Gleeson JF (2019). A pilot digital intervention targeting loneliness in youth mental health. Front Psychiatry.

[58] Alvarez-Jimenez M, Bendall S, Lederman R, Wadley G, Chinnery G, Vargas S, Larkin M, Killackey E, McGorry P, Gleeson J (2013). On the HORYZON: moderated online social therapy for long-term recovery in first episode psychosis. Schizophr Res.

[59] Chyzzy B, Nelson LE, Stinson J, Vigod S, Dennis C (2020). Adolescent mothers' perceptions of a mobile phone-based peer support intervention. Can J Nurs Res.

[60] Hawker S, Payne S, Kerr C, Hardey M, Powell J (2002). Appraising the evidence: reviewing disparate data systematically. Qual Health Res.

[61] DerSimonian R, Laird N (1986). Meta-analysis in clinical trials. Control Clin Trials.

[62] (2017). Stata Statistical Software: Release 15.

[63] Oxford English Dictionary Home. Oxford English Dictionary.

[64] (2013). Budgeting for involvement: Practical advice on budgeting for actively involving the public in research studies. National Institute for Health Research.

[65] Garrido S, Millington C, Cheers D, Boydell K, Schubert E, Meade T, Nguyen QV (2019). What works and what doesn't work? A systematic review of digital mental health interventions for depression and anxiety in young people. Front Psychiatry.

