# Supplementary material for: Quality Social Connection as an Active Ingredient in Digital Interventions for Young People With Depression and Anxiety: Systematic Scoping Review and Meta-analysis
Source: J Med Internet Res. 2021 Dec 17;23(12):e26584. doi: 10.2196/26584 (PMC8726025; doi:10.2196/26584)
Supplement: Multimedia Appendix 2 [file jmir_v23i12e26584_app2.docx]

**Multimedia Appendix 2: Step-wise approach to developing a full and objective search strategy**

| APA PsycInfo (1806 to 24^th^ June, 2020) | |
| --- | --- |
| 1 | Young people.mp |
| 2 | Young adult*.mp |
| 3 | Young person*.mp |
| 4 | Youth.mp |
| 5 | Adolescen*.mp |
| 6 | Teen*.mp |
| 7 | 1 or 2 or 3 or 4 or 5 or 6 [Establish population] |
| 8 | Social connect*.mp |
| 9 | Peer support.mp |
| 10 | Interaction.mp |
| 11 | Interpersonal.mp |
| 12 | Lonel*.mp |
| 13 | Social need*.mp |
| 14 | Sociali?ation.mp |
| 15 | Companionship.mp |
| 16 | Relatedness.mp |
| 17 | Sense of belonging.mp |
| 18 | Social belonging.mp |
| 19 | Closeness.mp |
| 20 | Social engagement.mp |
| 21 | Empath*.mp |
| 22 | Attachment.mp |
| 23 | *Empathy/ |
| 24 | Exp social support/ |
| 25 | Value* relation*.mp |
| 26 | Socio-relation*.mp |
| 27 | Social integration.mp |
| 28 | Social inclusion.mp |
| 29 | Compassion*.mp |
| 30 | 8 or 9 or 10 or 11 or 12 or 13 or 14 or 15 or 16 or 17 or 18 or 19 or 20 or 21 or 22 or 23 or 24 or 25 or 26 or 27 or 28 or 29 [Establish intervention – QSC] |
| 31 | Digital intervention.mp |
| 32 | Online*.mp |
| 33 | Digital*.mp |
| 34 | Internet*.mp |
| 35 | Web*.mp |
| 36 | Computer*.mp |
| 37 | Electronic*.mp |
| 38 | *Electronic Health Records/ |
| 39 | EMR.mp |
| 40 | HER.mp |
| 41 | Internet-support*.mp |
| 42 | Technology.mp |
| 43 | *Telemedicine/ |
| 44 | *Machine Learning/ |
| 45 | Email*.mp |
| 46 | Telehealth.mp |
| 47 | Telepsychi*.mp |
| 48 | Telemental.mp |
| 49 | e-mental.mp |
| 50 | Mhealth.mp |
| 51 | Avatar.mp |
| 52 | eLearn*.mp |
| 53 | Video*.mp |
| 54 | Virtual.mp |
| 55 | VR.mp |
| 56 | Mobile app*.mp |
| 57 | Mobile intervention* |
| 58 | Mobile phone*.mp |
| 59 | Smartphone*.mp |
| 60 | Android.mp |
| 61 | iPhone.mp |
| 62 | iOS.mp |
| 63 | 31 or 32 or 33 or 34 or 35 or 36 or 37 or 38 or 39 or 40 or 41 or 42 or 43 or 44 or 45 or 46 or 47 or 48 or 49 or 50 or 51 or 52 or 53 or 54 or 55 or 56 or 57 or 58 or 59 or 60 or 61 or 62 [Establish context – digital intervention] |
| 64 | *Anxiety/ or *anxiety disorders/ or exp depressive disorder/ |
| 65 | Depress*.mp |
| 66 | Mood disorder.mp |
| 67 | *depression/ |
| 68 | Anx*.mp |
| 69 | 64 or 65 or 66 or 67 or 68 [Establish mental health affect] |
| 70 | 7 and 30 and 63 and 69 |

Additional manually-searched sources:

| 1 | Horgan A, Sweeney J. Young students’ use of the internet for mental health information and support. J Psychiatr Ment Health Nurs 2010; 17: 117–23. |
| --- | --- |
| 2 | Sharabi A, Margalit M. Virtual friendships and social distress among adolescents with and without learning disabilities: The subtyping approach. *Eur J Spec Needs Educ* 2011; 26: 379–94. |
| 3 | Alvarez-Jimenez, *et al.* Enhancing social functioning in young people at Ultra High Risk (UHR) for psychosis: A pilot study of a novel strengths and mindfulness-based online social therapy. *Schizophr Res* 2018; 202: 369–77. |
| 4 | Cole DA, Nick EA, Zelkowitz RL, Roeder KM, Spinelli T. Online social support for young people: Does it recapitulate in-person social support; can it help? *Comput Human Behav* 2017; 68: 456–64. |
| 5 | Frison E, Eggermont S. Exploring the relationships between different types of Facebook use, perceived online social support, and adolescents’ depressed mood. *Soc Sci Comput Rev* 2016; 34: 153–71. |
| 6 | Mikami, AY, Szwedo DE, Allen JP, Evans MA, Hare AL. Adolescent Peer Relationships and Behavior Problems Predict Young Adults’ Communication on Social Networking Websites. *Dev Psychol* 2010; 46: 46–56. |
| 7 | Ozcan NK, Buzlu S. Internet use and its relation with the psychosocial situation for a sample of university students. *Cyberpsychol Behav* 2007; 10: 767–72. |
| 8 | Saulsberry A, Marko-Holguin M, Blomeke K, *et al.* Randomized Clinical Trial of a Primary Care Internet-based Intervention to Prevent Adolescent Depression: One-year Outcomes. *J Can Acad Child Adolesc Psychiatry* 2013; 22: 106–17. |
| 9 | Van Zalk MHW, Branje SJT, Denissen J, Aken MAG, Meeus. Who benefits from chatting, and why?: The roles of extraversion and supportiveness in online chatting and emotional adjustment. *Personal Soc Psychol Bull* 2011; 37: 1202–15. |
| 10 | Wright KB, Rosenberg J, Egbert N, Ploeger NA, Bernard DR, King, S. Communication competence, social support, and depression among college students: a model of facebook and face-to-face support network influence. *J Health Commun* 2013; 18: 41–57. |
| 11 | Yeh Y-C, Ko H-C, Wu JY-W, Cheng C-P. Gender differences in relationships of actual and virtual social support to Internet addiction mediated through depressive symptoms among college students in Taiwan. *Cyberpsychol Behav* 2008; 11: 485–7. |
| 12 | Van Zalk N, Tillfors M. Co-rumination buffers the link between social anxiety and depressive symptoms in early adolescence. *Child Adolesc Psychiatry Ment Health* 2017; 11: 41. |
| 13 | Bailey E, Alvarez-Jimenez M, Robinson J. *et al.* An enhanced social networking intervention for young people with active suicidal ideation: Safety, feasibility and acceptability outcomes. *Int J Environ Res Public Health* 2020; 17: 2435. |
| 14 | McCloskey W, Iwanicki S, Lauterbach D, Giammittorio DM, Maxwell K. Are Facebook ‘Friends’ Helpful? Development of a Facebook-Based Measure of Social Support and Examination of Relationships Among Depression, Quality of Life, and Social Support. *Cyberpsychol Behav Soc Netw* 2015; 18: 499–505. |
| 15 | Blackwell LS, Romero SL, Romero C V, *et al.* CFfone: A social networking site for adolescents and young adults with cf. *Pediatr Pulmonol* 2012; 47: 430. |
| 16 | Canady VA. Survey explores social media, mental well‐being among youth. *Ment Heal Wkly* 2018; 28: 3–4. |
| 17 | Feinstein BA, Bhatia V, Hershenberg R, Davila J. Another venue for problematic interpersonal behavior: The effects of depressive and anxious symptoms on social networking experience. *J Soc Clin Psychol* 2012; 31: 356–82. |
| 18 | Stockdale LA, Coyne SM. Bored and online: Reasons for using social media, problematic social networking site use, and behavioral outcomes across the transition from adolescence to emerging adulthood. *J Adolesc* 2020; 79: 173–83. |
| 19 | Colder Carras M, Van Rooij AJ, Van de Mheen D, Musci R, Xue Q-L, Mendelson T. Video Gaming in a Hyperconnected World: A Cross-sectional Study of Heavy Gaming, Problematic Gaming Symptoms, and Online Socializing in Adolescents. *Comput Human Behav* 2017; 68: 472–9. |
| 20 | Rice S, Gleeson J, Davey C, *et al.* Moderated online social therapy for depression relapse prevention in young people:  pilot study of a ‘next generation’ online intervention. 2018; 12: 613–25. |
| 21 | Rice S, O’Bree B, Wilson M, *et al.* Leveraging the social network for treatment of social anxiety: Pilot study of a youth-specific digital intervention with a focus on engagement of young men. *Internet Interv* 2020; 20: 100323. |
| 22 | Sharabi A, Margalit M. The mediating role of internet connection, virtual friends, and mood in predicting loneliness among students with and without learning disabilities in different educational environments. *J Learn Disabil* 2011; 44: 215–27. |
| 23 | Poppelaars M, Lichtwarck-Aschoff A, Kleinjan M, Granic I. The impact of explicit mental health messages in video games on players’ motivation and affect. *Comput Human Behav* 2018; 83: 16–23. |
| 24 | Felnhofer A, Kafka JX, Hlavacs H, Beutl L, Kryspin-Exner I, Kothgassner OD. Meeting others virtually in a day-to-day setting: Investigating social avoidance and prosocial behavior towards avatars and agents. *Comput Human Behav* 2018; 80: 399–406. |
| 25 | Liu CY, Yu CP. Can facebook use induce well-being? *Cyberpsychology, Behav Soc Netw* 2013; 16: 674–8. |
| 26 | Garrido S, Cheers D, Boydell K, *et al.* Young people’s response to six smartphone apps for anxiety and depression: Focus group study. *J Med Internet Res* 2019; 21. DOI:10.2196/14385. |
| 27 | Radovic A, DeMand AL, Gmelin T, Stein BD, Miller E. SOVA: Design of a stakeholder informed social media website for depressed adolescents and their parents. *J Technol Hum Serv* 2018; 35: 169–82. |
| 28 | Santesteban-Echarri O, Rice S, Wadley G, *et al.* A next-generation social media-based relapse prevention intervention for youth depression: Qualitative data on user experience outcomes for social networking, safety, and clinical benefit. *Internet Interv* 2017; 9: 65–73. |
| 29 | Selkie E, Adkins V, Masters E, Bajpai A, Shumer D. Transgender Adolescents’ Uses of Social Media for Social Support. *J Adolesc Heal* 2020; 66: 275–80. |
| 30 | Dolev-Cohen M, Barak A. Adolescents’ use of instant messaging as a means of emotional relief. *Comput Human Behav* 2013; 29: 58–63. |
| 31 | Bhuvaneswar CG, Gutheil TG. E-mail and psychiatry: some psychotherapeutic and psychoanalytic perspectives. *Am J Psychother* 2008; 62: 241–61. |
| 32 | Siriaraya P, Tang C, Ang CS, Pfeil U, Zaphiris P. A comparison of empathic communication pattern for teenagers and older people in online support communities. *Behav Inf Technol* 2011; 30: 617–28. |
| 33 | Dhesi M. A qualitative study to investigate in what ways are the distinctive features of synchronous text- based counselling experienced as being helpful and/or unhelpful by young people? — University of Roehampton Research Explorer. 2019. https://pure.roehampton.ac.uk/portal/en/studentTheses/a-qualitative-study-to-investigate-in-what-ways-are-the-distincti (accessed Sept 6, 2020). |
| 34 | Radovic A, Gmelin T, Stein BD, Miller E. Depressed adolescents’ positive and negative use of social media. *J Adolesc* 2017; 55: 5–15. |
| 35 | Horgan A, McCarthy G, Sweeney J. An Evaluation of an Online Peer Support Forum for University Students With Depressive Symptoms. *Arch Psychiatr Nurs* 2013; 27: 84–9. |
| 36 | Campbell A, Ridout B, Amon K, Navarro P, Collyer B, Dalgeish J. A Customized Social Network Platform (Kids Helpline Circles) for Delivering Group Counseling to Young People Experiencing Family Discord That Impacts Their Well-Being: Exploratory Study. *J Med Internet Res* 2019; 21: e16176. |
| 37 | Ellis LA, Campbell AJ, Sethi S, O’Dea BM. Comparative randomized trial of an online cognitive-behavioral therapy program and an online support group for depression and anxiety. *J CyberTherapy Rehabil* 2011; 4: 461–7. |
| 38 | Lim MH, Rodebaugh TL, Eres R, Long KM, Penn DL, Gleeson JFM. A Pilot Digital Intervention Targeting Loneliness in Youth Mental Health. *Front Psychiatry* 2019; 10: 604. |
| 39 | Alvarez-Jimenez, Bendall S, Lederman R, *et al.* On the HORYZON: Moderated online social therapy for long-term recovery in first episode psychosis. *Schizophr Res* 2013; 143: 143–9. |
| 40 | Clarke C S. Telepsychiatry in Asperger’s syndrome. *Ir J Psychol Med* 2018; 35: 325–8. |
| 41 | Chyzzy B, Nelson LE, Stinson J, Vigod S, Dennis C. Adolescent Mothers’ Perceptions of a Mobile Phone-Based Peer Support Intervention. *Can J Nurs Res* 2020; 52: 129–38. |
| 42 | van Rensburg SH, Klingensmith K, McLaughlin P, Qayyum Z, van Schalkwyk GI. Patient-provider communication over social media: perspectives of adolescents with psychiatric illness. *Heal Expect* 2016; 19: 112–20. |
