# Supplementary material for: Quality Social Connection as an Active Ingredient in Digital Interventions for Young People With Depression and Anxiety: Systematic Scoping Review and Meta-analysis
Source: J Med Internet Res. 2021 Dec 17;23(12):e26584. doi: 10.2196/26584 (PMC8726025; doi:10.2196/26584)
Supplement: Multimedia Appendix 3 [file jmir_v23i12e26584_app3.pdf]

Multimedia Appendix 3: Expanded data extraction table and quality assessment

Author/year/ country/quality	Study design	Setting/ Participants	Pathway stage	Digital intervention	QSC indicator(s)/ mechanism(s)/ measure(s)	Outcome(s)/ measure(s)	Results
Alvarez-Jimenez et al. (2013). Australia. High quality	Quantitative and qualitative, uncontrolled single-group, observation, questionnaire, semi-structured interview	Setting: Early psychosis prevention and intervention centre Sample: 20 patients (50% female; 15-25 years old; 45% Anglo Australian, 25% Asian, 10% bi-racial and 5% African) Presenting condition: First episode psychosis	Treatment	Peer-to-peer online social networking, individually tailored interactive psychosocial interventions, expert moderation - HORYZONS	Indicator(s): Social connectedness Mechanism(s): Moderation Measure(s): User Experience approach-based questionnaire and semi-structured interview	Outcome(s): Depression and anxiety Measure(s): BPRS, CDRS, BAI	All clinical measures improved: BPRS at baseline M(SD) 33.75(7.70), and at 1 month follow up 33.45(8.24) p=0.8. CDRS at baseline 4.85(5.95), and at follow up 3.55(5) p=0.02 D=0.60. BAI at baseline 15.95(10.87), and at follow up 12.8(11.81) p=0.15. . Moderation considered supportive by 90%. 60% reported significantly increased social connectedness.
Alvarez-Jimenez et al. (2018). Australia. Medium quality	Quantitative, uncontrolled single-group, observation, semi-structured interview	Setting: PACE clinic for ultra-high risk psychosis Sample: 14 patients (79% female; 15-25 years old; Ethnicity unknown, all Australia-born) Presenting condition: Ultra high risk for psychosis	Treatment	Online social networking, peer-to-peer and professional moderation - MOMENTUM	Indicator(s): Social support, loneliness Measure(s): SPS and UCLA Loneliness Scale	Outcome(s): Depression and psychological wellbeing Measure(s): SWLS, MADRS, PSS	Correlations between perceived social support and depressive symptoms (rs=−0.49; p=0.08) and loneliness (rs=−0.48; p=0.09). Improved depression ratings from baseline M(SD) 18.33(15.3) to 12 week follow up 13(9.01) d=0.3, p=0.11. Improved perceived stress from baseline 24.33(8.14) to follow up 21.83(6.82) d=0.5, p=0.33. Improved subjective wellbeing from baseline 15.33(5.1) to follow-up 18.25(6.55) d=0.75, p=0.03. 33% reliable decline in loneliness. Statistically significant increases from baseline to follow-up in subscales of social support.
Bailey et al. (2020). Australia. High quality	Quantitative, uncontrolled single-group pre-/post-test, observation, semi-	Setting: Tertiary-level mental health service Sample: 20 patients (55% female; 16-25 years old; Ethnicity	Treatment	Enhanced online social networking intervention - Affinity	Indicator(s): Social connectedness, feeling you are not a burden, sense of belonging Measure(s): Self-report using SCS-R and	Outcome(s): Depression Measure(s): PHQ-9	Significant improvement in PHQ-9 scores from baseline M(SD) 19.6(4.1), to 8 week intervention 15.5(6.9) d=-0.94 p=0.016. INQ-PB score at baseline

	structured interview	unknown, country of birth 75% Australia, 20% Asia, 5% UK) Presenting condition: Suicidal ideation			perceived burdensomeness and thwarted belongingness via self-report INQ-15		26.7(10.3) and post-intervention 21.6(11.1) d=-.52 p=0.048. INQ-TB score at baseline 44.1(10.0) and post-intervention 35.2(14.4) d=-0.96 p=0.006. SCS at baseline 21.6(9.5), and post-intervention 27.9(12.2) d=-.54 p=0.062 .
Bhuvaneswar & Gutheil (2008). USA. High quality	Qualitative , retrospective case study, observation	Setting: Psychodynamic psychotherapy clinic Sample: 1 patient (Female; 17 years old; Ethnicity unknown) Presenting condition: Depression	Treatment	Instant messenger	Indicator(s): Negative social interaction, feeling ignored Measure(s): Self-reported mood	Outcome(s): Psychological wellbeing Measure(s): Self-report	Hurt and scared because of being ignored by therapist on social media.
Blackwell et al. (2012). USA. High quality	Quantitative, RCT, questionnaire	Setting: General Sample: 100 adolescents (62% female; mean age 15.69 years old; 57% Caucasian, 16% Hispanic, 9% African American, 18% Ethnicity unknown) Presenting condition: Cystic fibrosis	Treatment and prevention	Online social networking peer support programme - CFfone.com	Indicator(s): Social support Measure(s): SNS, CFQ-R	Outcome(s): Depression and anxiety Measure(s): HADS	Those who felt more supported by friends had lower depression (r=-.26, p=<.01) and anxiety symptoms (r=-.19, p=.04), and higher Social Functioning scores (r=.22, p=.02). Those with a mobile phone at baseline also reported higher Social Functioning scores on the CFQ-R (T=2.3, p=.02)
Campbell et al. (2019). Australia. Medium quality	Qualitative and quantitative, participatory action research design, observation, questionnaire	Setting: Kids Helpline family discord service Sample: 105 callers of helpline (82% female; 13-25 years old; Ethnicity unknown) Presenting condition: Mild-to-moderate depression or anxiety (not high risk)	Treatment	Social networking site for peer-to-peer and counsellor-to-peer group support	Indicator(s): Relatedness, social connectedness, less alone in one’s feelings, Mechanism(s): Supporting others Measure(s): MSPSS and thematic analysis	Outcome(s): Depression and anxiety Measure(s): CES-D, RCMAS	Data quality pertaining to anxiety and depression outcomes too low to conduct meaningful analysis (due to drop-off in response rates) The main benefits participants hoped to gain from the site were engaging with others they could relate to due to similar lived experience, to not feel alone, to connect with others, and to engage with others for support. Participants also hoped to provide support to others.

Canady (2018). USA. High quality.	Quantitative, cross-sectional study, questionnaire, interview	Setting: General Sample: 1300 adolescents (Gender unknown; 14-22 years old; Ethnicity unknown) Presenting condition: None in particular	Treatment and prevention	Online health information, digital health tools in general including peer-to-peer health exchange networks	Indicator(s): Less alone in one's feelings, connecting with similar people Mechanism(s): Comparison to others' experiences Measure(s): PHQ-9 and thematic analysis of interviews	Outcome(s): Depression and anxiety Measure(s): PHQ-9 and self-report	More teens and young adults say using social media when they are depressed, stressed or anxious makes them feel better (27%) than say it makes them feel worse (15%). 20% say it connects them to helpful support and advice, compared to 15% who say it sends them "down a rabbit hole". 75% of respondents looked at other people's stories to understand and learn so that they would know 'I'm not alone in this,'". 39% of young people say they try to find people with similar health conditions online. 91% of those who found health peers online say the experience was at least "somewhat" helpful.
Chyzzy et al. (2020). Canada. High quality.	Qualitative and quantitative, uncontrolled single-group design, questionnaire, semi-structured interview	Setting: MPPS intervention group Sample: 21 mothers (100% female; 17-24 years old, mean age 21.3 SD=1.8; Ethnicity unknown, country of birth 66.7% Canada) Presenting condition: Generally healthy, 14.3% with prior history of depression	Prevention	Individualised peer mentor support via phone call and text messaging - MPPS intervention	Indicator(s): Trust, loneliness, feeling accepted, feeling normalised and close to peer Mechanism(s): Anonymity Measure(s): PSEI	Outcome(s): Depression and anxiety Measure(s): Self-report	68.8% felt less depressed after intervention – 27% attributable to their peer mentor. 50% felt less worried/anxious - 50% attributable to their peer mentor. 43.4% of mothers who reported improvements in stress and coping attributed them to help from their peer mentor. 43.9% enjoyed the anonymity. 93% considered their peer trustworthy, 100% felt accepted and 75% felt normalised. 50% felt close to their peer. 75% felt less lonely.
Clarke (2018). Ireland. High quality.	Qualitative, retrospective case study, observation	Setting: Clinical Sample: 1 patient (Male; 16 years old; Ethnicity unknown)	Treatment	Telepsychiatry	Indicator(s): Rapport Measure(s): Clinician observation	Outcome(s): Treatment engagement Measure(s): Observation	Telepsychiatry offered a gateway to face-to-face therapy. At baseline, patient refused to attend therapy in person, and after 4-5 months of telepsychiatry

		Presenting condition: Asperger's with comorbid depression					sessions, rapport was established between the patient and the provider, and the patient agreed to a face-to-face appointment
Colder Carras et al. (2017). USA. Medium quality	Quantitative, cross-sectional study, questionnaire	Setting: 30 USA schools Sample: 9733 students (51% female; 13-16 years old, average age 14.1; 82.1% Dutch) Presenting condition: None in particular	Treatment and prevention	Online video gaming	Indicator(s): Social connectedness Measure(s): NRI	Outcome(s): Depression and social anxiety Measure(s): Depressive mood list, SASC-R	Males: Lower depression levels found in Social Engaged gamers (b=0.40 SE=0.12 p=0.00) compared to problematic gamers (1.21 0.32 0.00), at-risk gamers (0.81 0.08 0.00) social at-risk (0.85 0.17 0.00) and extensive gamers (0.55 0.06 0.00) and this group also had lowest level of low online/offline friendship quality. Social Engaged gamers had lowest social anxiety levels (0.21 0.11 0.05) compared to (0.24 0.23 0.29), (0.20 0.07 0.00), (0.11 0.15 0.46), (0.40 0.06 0.00) respectively for the above groups of gamers. Females: the same trends are noted (with different numbers).
Cole et al. (2017). USA. N/A.	Quantitative, uncontrolled single group design, questionnaire	Setting: Private university Sample: 231 undergraduate students (72% female; average 19.28 years old SD=1.15; 67.1% Caucasian, 23.4% Asian American, 10.4% African American, 5.2% Hispanic/Latino, 0.4% Other) Presenting condition: None in particular	Treatment and prevention	Online social networks in general	Indicator(s): Social support Measure(s): SNS2, PSSS	Outcome(s): Depression Measure(s): DASS, CTI, BDI-II	For online social support, effect on depressive thoughts and feelings (RSE, BDI, CTI, DAS loaded into single latent DTE factor): β=0.16, z=2.10 p<0.05. This effect is weaker than for in-person support.
Dhesi (2019). UK. High quality.	Qualitative, cross-sectional, online semi-structured interviews	Setting: Kooth Sample: 13 Kooth users (69% female; 14-18 years old;	Treatment	Online counselling (text)	Indicator(s): Social connectedness, feeling normalised, being able to share	Outcome(s): Anxiety Measure(s): Thematic	Reported benefits of using online counselling: Reduced distress and anxiety, sharing more, increased

		69.2% White British, 15.4% White Asian, 15.4% Other) Presenting condition: None in particular			Mechanism(s): Anonymity, disinhibition effect Measure(s): Thematic analysis of semi-structured interviews	analysis of interviews	disinhibition effect, social connectedness, normalisation, empowered. Unhelpful factors of online counselling included not getting desired help, unable to express self.
Dolev-Cohen & Barak (2013). Israel. High quality.	Qualitative, case-control design, questionnaire, textual analysis, observation	Setting: General Sample: 150 Instant Messaging users (63% female; 14-18 years old, Ethnicity unknown) Presenting condition: Distressed vs non-distressed groups of participants	Treatment and prevention	Regular use of Instant Messaging	Indicator(s): Emotional connection Measure(s): Thematic analysis of conversations	Outcome: Psychological wellbeing Measure: PANAS	Emotional distress of distressed participants decreased from 28.74 to 22.83, while there was no significant change in non-distressed participants. Experts' ratings of the level of emotional distress of the distressed participants was M(SD) 2.69(0.83) in the first half of a chat and 3.41(0.75) in the second half. The improvement in emotional state was found to be significant (F=46.06; df=98; p<.001; g2=0.32). For relationship between introversion-extraversion and different levels of distress improvement: r=-0.19, p<0.05, Pearson correlation with self-reported distress, r=0.08 with emotional negative expressions, and r=0.02 with judges' ratings of distress level.
Ellis, Campbell, Sethi, & O'Dea (2011). Australia. N/A.	Qualitative and quantitative, comparative randomised controlled trial, questionnaire	Setting: University students not receiving mental health treatment Sample: 39 students (77% female; 18-25 years old, mean age 19.67 SD=1.66; Ethnicity unknown) Presenting condition: Anxiety and/or depression, but none severe	Treatment	Online cognitive behaviour therapy (CBT) self-help program (MoodGYM) compared with online support group (MoodGarden)	Indicator(s): Social support, relatedness Mechanism(s): Anonymity Measure(s): OSSS	Outcome: Depression, anxiety, and psychological wellbeing Measure: DASS, ATQ	Both online CBT and online peer support were effective in reducing anxiety compared with the control condition which was no treatment (online CBT versus control: t=-2.26, p=.03; online peer support versus control: t=-2.64, p=.01). Online social support significantly improved for online peer support group compared with online CBT and control

							(online peer support versus control: t=2.31, p=.03; online peer support versus online CBT: t=3.62, p<= .001). Larger reduction in DASS depression and anxiety scores from baseline to follow up in online support group than online CBT or control. Participants in the peer support group valued being able to relate to others with similar experiences.
Feinstein, Bhatia, Hershenberg, & Davila (2012). USA. High quality.	Quantitative, short-term prospective cohort study, questionnaire	Setting: Undergraduate university students Sample: 301 students (62% female; mean age 19.44 years old SD=2.05; 41% Asian/Pacific Islander, 41% Caucasian, 6% Latino 6% African American, 6% Other) Presenting condition: Some participants had raised depression/ anxiety/ social anxiety at baseline	Treatment and prevention	Social networking in general	Indicator(s): Negative interactions Measure(s): SNS3	Outcome: Depression and anxiety (including social anxiety) Measure: DASS, BFNE	Depressive symptoms at time 1 predicted more negative interactions with close friends and romantic partners and less positive interactions with romantic partners only. Additionally, depressive symptoms at time 1 predicted increases in depressed and anxious affect following interactions with close friends, romantic partners, and people in general. Global anxiety symptoms did not predict any of the social networking interaction quality variables.
Felnhofer et al. (2018). Austria. N/A.	Quantitative, RCT, questionnaire	Setting: Public university Sample: 95 students (87% female; mean age 23.34 years old SD=2.727; Ethnicity unknown) Presenting condition: None in particular	Prevention	Avatars (virtual entities controlled by another human being) and agents (virtual entities controlled by a computer)	Indicator(s): Empathy Measure(s): NMMSP	Outcome: Anxiety Measure: SIAS	Participants who talked to avatars M(SD) 36.19(7.353) had similar levels of social interaction anxiety to those who interacted with agents 35.37(6.850), t(93)=-0.560, p=0.577. Participants in the avatar group showed significantly higher empathy levels 21.72(5.926) than participants in the agent group 17.646(7.431), t(93)=-2.953, d=0.61, p=0.004 .

Frison & Eggermont (2016). Belgium. Medium quality.	Quantitative, uncontrolled cross-sectional, questionnaire	Setting: 18 randomly selected high schools in Flanders, Belgium Sample: 910 students with Facebook account (52% female; average age 15.44 years old SD=1.71; Ethnicity unknown, country of birth 96.1% Belgium, 1.8% Europe, 2.1% non-European country) Presenting condition: None in particular	Treatment and prevention	Facebook	Indicator(s): Social support Measure(s): Family subscale of MSPSS	Outcome: Depression Measure: CES-DC	The relationship between active private Facebook use and adolescents' depressed mood was significantly mediated by perceived online social support (POSS), CI: [-.032,-.002], SE=.01, $p<.01$. POSS also mediated the relationship between active public Facebook use and depressed mood, CI: [-.043, -.003], SE=.01, $p=.01$. Active private Facebook use positively predicted girls', $\beta=0.27$, B=0.20, SE=.04, $p<.001$, but not boys' POSS. The path-by-path analysis further confirmed that the relationship between private Facebook use and adolescents' POSS significantly differed between boys and girls ($p<.05$). Finally, results showed that POSS negatively predicted girls' depressed mood, β =0.12, B=-0.05, SE=0.02, $p<.05$, but not boys' depressed mood.
Garrido et al. (2019). Australia. Medium quality.	Qualitative, focus groups	Setting: High schools and universities in Western Australia Sample: 23 students (65% female; 13-25 years old; Ethnicity unknown) Presenting condition: DASS score <15 (severely depressed excluded)	Treatment and prevention	Six currently available smartphone apps for mental health (Mood Mission, Music eScape, Pacifica, Mindshift, Headspace, and What's Up)	Indicator(s): Negative interactions, social connectedness, relatedness Mechanism(s): Anonymity, personalisation, conjunct face-to-face support Measure: Thematic analysis of focus group content	Outcome: Helpful and unhelpful aspects of smartphone apps for mental health Measure: Thematic analysis of focus group content	Of six main themes identified that were important features of the apps, one was social connection, another personalisation. Some valued anonymity, while others worried that some users would use anonymity to be hurtful on forums. Some concerns that forums would need to be highly moderated to avoid harm. Benefits of peer support included knowing others are sharing the same issues as

							you. Some expressed concern that apps could be used as a crutch, and people use them to avoid face-to-face contact with people.
Horgan, McCarthy & Sweeney (2013). Ireland. Medium quality.	Qualitative and quantitative, pre-post test and qualitative descriptive designs, extraction of posts from website, questionnaire for CES-D scores	Setting: University of Cork Sample: 118 students (36% female; 18-24 years old; 98.3% White, 1.7% Asian or Asian Irish) Presenting condition: Depression	Treatment	Depression support website (www.losetheblues.ie) with peer support forum	Indicator(s): Less alone in one's feelings, being able to share, shared understanding Measure(s): Thematic analysis of posts on peer support forum	Outcome: Depression Measure: CES-D	Only 16 individuals completed the post-test questionnaire, of which 81% were male. The median (IQR) CES-D score was 37.00(33.0-43.25) at baseline and 33.50(22.25-40.50) post-intervention. While a difference can be noted it was not statistically significant (p=0.133). Benefits of the peer support forum were feeling less alone, being able to share feelings, and knowing that others are feeling the same, and that they have a shared understanding of how they feel.
Horgan & Sweeney (2010). Ireland. Medium quality	Quantitative, descriptive study, questionnaire	Setting: University Sample: 922 students (62% female; 18-24 years old, Ethnicity unknown) Presenting condition: None in particular	Treatment and prevention	Internet use for mental health support	Indicator(s): Connecting with similar people Mechanism(s): Anonymity, confidentiality, knowing who produced information Measure(s): 30-item self-designed questionnaire	Outcome: Reasons for use of internet-based mental health support Measure: Self-developed questionnaire	30.8% used the internet for mental health information, 22.3% said this was because it's anonymous, private and confidential, 10.1% because it's easily accessible, 3.7% because it's easy to communicate with other young people in the same situation. When asked what they deemed to be important in terms of website design and content if they were to use the internet for mental health support, the most important issues identified were confidentiality, anonymity, being able to ask questions, usability and knowing who produced the information.

Lim et al (2019). Australia. High quality.	Qualitative and quantitative, descriptive design, pre-post test questionnaires, mood tracker, semi-structured interview	Setting: Local youth health service (SAD participants) and Australian university (non-SAD participants) Sample: 20 participants (45% female; 18-23 years old; 91% White, 9% Multiracial or other) Presenting disorder: With or without social anxiety disorder (SAD)	Treatment and prevention	+Connect, a digital smartphone application with video material	Indicator(s): Social connectedness, feeling close to a peer Measure(s): Thematic analysis from semi-structured interview	Outcome: Depression and anxiety Measure: CES-D, SIAS	6/8 in SAD group felt satisfied that the intervention helped them feel closer to others, and in the non-SAD group 9/11 felt this way. For the non-SAD group,S-SIAS and CES-D scores decreased from baseline (M(SD) 29.18(7.85) and 11.55(7.10) respectively) to post-intervention (21.64(14) and 8.45(7.37) respectively) and 3 months post intervention (22(11.96) and 8.45(6.93) respectively). For the SAD group. S-SIAS scores decreased in a linear trend from baseline (43.22(7.56)) to 3 months post-intervention (34.89(13.8)), however, CES-D scores decreased from baseline (21.89(7.75)) to posttreatment (14(5.51)), but scores regressed toward baseline at 3-months post-intervention (15.56(8.88)). Participants reported finding the real-life application of social skills both rewarding and challenging.
Liu & Yu (2013). Taiwan. Medium quality.	Quantitative, cross-sectional study, questionnaire	Setting: College Sample: 330 Facebook-using students (63% female; 18-23 years old; Ethnicity unknown) Presenting condition: None in particular	Prevention	Facebook	Indicator(s): Social support Mechanism(s): Accessibility, conjunct face-to-face support Measure(s): ISEL, modified for online use	Outcome: Psychological wellbeing Measure: Ryff's scales of psychological wellbeing	Intensity of Facebook use significantly predicted online social support (β=0.481, $p<0.001$, R^2 =0.231). Online social support was significantly related to general social support (β=0.424, $p<0.001$, R^2 =0.180). The direct effect between online social support and wellbeing was β=0.095 (t=1.738, $p<0.05$). The test results of mediation analyses show that the relationship

							between online social support and well-being is mediated through general social support.
McCloskey, Iwanicki, Lauterbach, Giammittorio, & Maxwell (2015). USA. Medium quality.	Quantitative, uncontrolled single-group design, questionnaire	Setting: University Sample: 633 Undergraduate students with Facebook page (70% female; minimum age 18, median age 21; 64.8% Caucasian) Presenting condition: None in particular - participants on average had mild levels of depression at baseline	Treatment and prevention	Facebook	Indicator(s): Social support Measure(s): FMSS, MSPSS, ISSB	Outcome: Depression Measure: PHQ-9	Perceived social support was negatively associated with depression severity (-0.204 p<0.01). Received social support was not significantly related to depression but was positively related to psychological domain of Quality of Life (0.108 p<0.05).
Mikami, Szwedo, Allen, Evans, & Hare (2010). USA. High quality.	Quantitative, longitudinal, observation, questionnaire	Setting: Public middle school Sample: 92 social networking site users (58% female; mean age 20.92 years old SD=1.11; 58% White, 29% African American, 13% Other or Mixed) Presenting condition: None in particular	Treatment and prevention	Online social networking	Indicator(s): Social support Measure(s): Dimensions and Types of Social Status observed positivity and negativity in peer interaction	Outcome: Depression Measure: CDI	No significant depression outcomes. The only demographic variable that predicted support was gender, such that female participants, in comparison with male participants, had significantly more friends posting highly supportive comments on their web page.
Ozcan & Buzlu (2007). Turkey. High quality.	Quantitative, uncontrolled single-group design, questionnaire	Setting: University Sample: 730 Undergraduate students that use the internet (53% female; mean age 20.84 years old SD=1.95; Ethnicity unknown) Presenting condition: None in particular	Treatment and prevention	Internet use in general	Indicator(s): Social support Measure(s): MDPSS	Outcome: Depression Measure: BDI	Statistically significant effect of MSPSS on BDI (β=-0.266) p<0.001.
Poppelaars, Lichtwarck-Aschoff, Kleinjan & Granic (2018). The Netherlands. Medium quality.	Quantitative, RCT, questionnaire	Setting: University Sample: 146 Undergraduate students that play video games (71% female; mean age	Treatment and prevention	Video game including cooperation with other players and with mental health messaging vs	Indicator(s): Relatedness Measure(s): Player Experience of Need Satisfaction scale	Outcome: Depression and psychological wellbeing Measure: BDI-II, SAM,	No difference in relatedness experienced in mental health messaging group vs non-mental health messaging group. Those with higher depressive

		20.2 years old SD=1.74; Ethnicity unknown, nationality 76% Dutch, 23% German, 1% Other) Presenting condition: None in particular - some with higher depressive symptoms at outset		without mental health messaging		International PANAS short form	symptoms experienced a higher level of relatedness to the other player (R^2=0.03, p=0.04). Positive affect increased across all participants (Total(SD) 14.05(3.43) to 16.74(4.17)), while the increase was larger for those higher in depressive symptoms.
Radovic, DeMand, Gmelin, Stein, & Miller (2017). USA. High quality.	Qualitative, RCT, semi-structured interviews, think aloud, advisory boards, focus groups	Setting: Academic adolescent medicine clinic and specialty psychiatry clinic Sample: 23 patients (78% female; 13-20 years old, mean age 16, SD=2.3) Presenting condition: Depression	Treatment	Social media website for depressed adolescents	Indicator(s): Social support Mechanism(s): Supporting others Measure(s): Thematic analysis from semi-structured interviews	Outcome: Adolescent-informed design of social media website for depression Measure: Thematic analysis from semi-structured interviews	Adolescents informed the design of the social media website for depression - aspects relating to desire for social connection via this platform are noted. Stakeholders wanted to provide peer support to other adolescents in similar situations, and reported feeling supported by hearing about others' experiences
Radovic, Gmelin, Stein, & Miller (2017). USA. Medium quality.	Qualitative, uncontrolled cross-section study, semi-structured interview	Setting: Academic adolescent medicine clinic and specialty psychiatry clinic Sample: 23 patients (78% female; 13-20 years old, mean age 16, SD=2.3; 87% Caucasian) Presenting condition: Depression	Treatment and prevention	Social media	Indicator(s): Connecting with similar people, feeling accepted, negative interactions Mechanism(s): Distraction from symptoms Measure(s): Thematic analysis from semi-structured interviews	Outcome: Depression and psychological wellbeing Measure: Thematic analysis from semi-structured interviews	Participants described mood improvements as a result of using SM. When feeling lonely adolescents could find acceptance online by connecting with similar people. Some avoided SM when depressed to avoid bringing others down with their negative mood. Others liked to use SM as distraction from their feelings when depressed.
Rice et al. (2018). Australia. Medium quality.	Quantitative, uncontrolled single-group pilot, structured clinical interview, questionnaire	Setting: Mental health clinic Sample: 42 patients (50% female; 15-25 years old, mean age 18.5, SD=2.1; Ethnicity unknown, country of birth 95.2% Australia) Presenting condition:	Treatment and prevention	Novel, moderated online social therapy intervention - Rebound	Indicator(s): Social connectedness, feeling accepted Measure(s): MOSSS, 2-Way Social Support Scale	Outcome: Depression and anxiety Measure: MADRS, DASSS	95% of participants thought Rebound was helpful for feeling more socially connected. All participants felt accepted by the forum moderators. Significant improvement in MADRS from baseline (α=0.73 M(SD) 16.2(6.9) to 12 week follow up (α=0.93 12.1(11.4) d=0.45 p=0.014. No

		Previous depression sufferers					significant improvement in anxiety scores, social connectedness, or social support.
Rice et al. (2020). Australia. High quality.	Quantitative, single group uncontrolled pre-post design, questionnaire	Setting: Four Headspace early intervention centres in north-western Melbourne Sample: 89 patients (47% female; 14-25 years old; Ethnicity unknown) Presenting condition: Social anxiety	Treatment	Social networking platform for socially anxious young people (Entourage) - a 'wall' function allows posting, commenting publicly	Indicator(s): Social connectedness, sense of belonging, feeling you are not a burden, loneliness Measure(s): DSSI, UCLA Loneliness Scale, SoCS, INQ	Outcome: Depression, social anxiety and psychological wellbeing Measure: PHQ-9, MDRS-22, LSAS, BFNE, SIAS, SWEMWBS	Depression, wellbeing and social anxiety scores improved for whole group from baseline to 12 week post treatment. PHQ-9: baseline M(SD) 13.74(5.63) to follow-up 10.52(5.69) p=<0.001. MDRS-22: 40.17(19.4) to 35.56(21.13) p=0.01. LSAS: 85.4(23.72) to 73.65(25.62) p=<0.001. BFNE: 50.3(9.66) to 47.07(10.46) p=0.001. SIAS: 54.75(11.06) to 49.46(13.47) p=<0.001. SWEMWBS: 19.58(3.69) to 21.82(4.41) p=<0.001. SCoS: 62.96(16.22) to 72.11(18.74) p<0.001. INQ-burden: 16.42(8.95) to 12.81(8.33) p<0.001. INQ-thwarted belonging: 36.79(9.66) to 31.23(11.98) p<0.001. UCLA: 53.95(9.90) to 48.39(10.58) p<0.001. DSSI: 28.64(5.67) to 31.37(6.28) p<0.001. Males showed reliable improvement on 14/22 variables, and non-males on 18/22 variables.
Santesteban-Echarri et al. (2017). Australia. Medium quality.	Qualitative, uncontrolled single-group pilot, semi-structured interview, focus group data	Setting: Mental health clinic Sample: 42 patients (50% female; 15-25 years old, mean age 18.5, SD=2.1; Ethnicity unknown, country of birth 95.2% Australia) Presenting condition: Previous depression sufferers	Prevention	Novel, moderated online social therapy intervention - Rebound	Indicator(s): Social support, relatedness Mechanism(s): Personalisation Measure(s): Thematic analysis of qualitative results	Outcome: Efficacy and usability evaluation of online social therapy intervention Measure: Thematic analysis from semi-structured interviews	Because peer supporters were in recovery themselves, they created a feeling of hope, and their advice was taken with more credibility. They were seen as a role model to follow. The moderators were viewed as inclusive and their comments were personalised and relevant. Their availability and constant interaction

							maintained the friendly and helpful tone of the site while encouraging participation from young people, increasing engagement. Most helpful component of intervention (% agree): social networking (57.9%), therapy (31.6%) personalised content from moderators (26.3%).
Saulsberry et al (2012).<br>USA.<br>Medium quality.	Quantitative, RCT, telephone interview	Setting: 12 Primary care sites across Southern and Midwestern United States<br>Sample: 58 patients (57% female; mean age 17.26 years old SD=1.85; 61% White, 24% Black, 6% Asian, 5% Hispanic, 4% Other)<br>Presenting condition: Depression	Prevention	PCP motivational interview (MI) + CATCH-IT Internet Program vs PCP brief advice (BA) + CATCH-IT Internet Program	Indicator(s): Loneliness<br>Measure(s): Positive Relationships Physician Scale	Outcome: Depression<br>Measure: CESD-10, DSM-IV-TR, PHQ-A	MI and BA groups had similar patterns and significant declines in mean CESD-10 scores (MI: ES=0.84, 95% CI: 0.39, 1.27; BA: ES=1.08, 95% CI: 0.60, 1.54) as well as the percentage of those with clinically significant depression symptoms from baseline to one-year follow-up. In both groups PHQ-A scores declined significantly from baseline to one-year follow-up (MI: ES=1.02 (95% CI: 0.56, 1.46); BA: ES=0.64 (95% CI: 0.19, 1.09). More participants in the BA group had experienced a depressive episode by the one-year follow-up (32.5% vs. MI: 11.6%, p=0.03, In MI there was a significant decline in the mean loneliness score both from baseline to one-year follow-up (ES=0.43, 95% CI: 0.00, 0.85) and from six-weeks to one-year follow-up (ES=0.36, 95% CI: -0.07, 0.79), while this significance pattern was only seen in the BA group from baseline to one-year follow-up (ES=0.54, 95% CI: 0.08, 0.98).

Selkie, Adkins, Masters, Bajpai, & Shumer (2020). USA. High quality.	Qualitative, uncontrolled single-group design, semi-structured interviews	Setting: Paediatric gender clinic Sample: 25 Transgender adolescents with social media profile (44% transfeminine; 15-18 years old, mean age 16; 80% White non-Hispanic, 4% African American, 8% American Indian, 8% Asian) Presenting condition: None in particular	Prevention	Social media platforms including YouTube, Instagram, Facebook, Twitter, Tumblr	Indicator(s): Connecting with similar people, loneliness, feeling validated, feeling normalised, negative interactions Measure(s): Thematic analysis of qualitative results	Outcome: Positive and negative outcomes of using social media for mental health support Measure: N/A	Participants described SM as a place to connect with similar (transgender) people. This was especially important to participants who did not feel that people they knew offline would understand what they were going through. SM were described as helpful in decreasing feelings of isolation. Participants described feeling validated and normalised by transgender-related SM content, particularly when the person posting the content was a public figure. Although SM were an overall positive tool for support among participants, negative aspects of SM were also identified. More than one in four participants mentioned instances of personal harassment.
Sharabi & Margalit (2011a). Israel. Medium quality.	Quantitative, cross-sectional crossover, questionnaire	Setting: Middle to high socioeconomic families vs those who failed in school (mostly from low socioeconomic families) Sample: 716 students (48% female; 16-18 years old; Ethnicity unknown) Presenting condition: With or without learning disabilities	Prevention	Internet communication	Indicator(s): Loneliness Measure(s): Hebrew adaptation of Loneliness Scale, ICQ	Outcome: Psychological wellbeing Measure: Hebrew adaptation of Mood Scale	Those with higher negative mood scores had stronger loneliness feelings (β=0.39 p=<0.01) and those with higher positive mood scores reported less intense loneliness feelings (β=-0.52 p=<0.01). Students with higher levels of Internet communication (with people they knew offline) reported less intense feelings of loneliness (β= -.08 p=<0.05), whereas students with higher levels of virtual friendships (with people that they met online) reported stronger feelings of loneliness (β=.13 p=<0.01).
Sharabi & Margalit (2011b). Israel.	Quantitative, cross-sectional case-control	Setting: 3 High schools in urban Israel	Prevention	Internet communication	Indicator(s): Loneliness Measure(s): Hebrew adaptation of	Outcome: Psychological wellbeing	For the whole group, virtual friendship (with people they met online) was positively

Medium quality.		Sample: 887 students grade 10-12 (50% female; 16-18 years old; Ethnicity unknown) Presenting condition: With (n=213) or without (n=674) learning disabilities (LD)			Loneliness and Social Dissatisfaction Scale, Internet Scale	Measure: Hebrew adaptation of Affect Scale	related to negative affect (β=0.16 $p<0.01$), and negatively related to positive affect (β=-.11 $p<0.01$). Internet communication (with people they knew offline) was positively related to negative affect (β=0.12 $p<0.01$) and was not significantly related to positive affect. Students with LD expressed higher levels of loneliness and negative affect. No significant differences were found for internet communication, virtual friendships and positive affect between LD and non-LD group.
Siriaraya, Tang, Ang, Pfeil, & Zaphiris (2011). Cyprus. Medium quality.	Qualitative, cross-sectional study, content analysis	Setting: General Sample: 400 messages from teenagers using online discussion forum (Gender unknown; Age range unknown; Ethnicity unknown) Presenting condition: None in particular	Treatment and prevention	Online anonymous discussion forum TeenHelp.org	Indicator(s): Emotional connection Measure(s): Thematic analysis of conversations	Outcome: Level of support provided between adolescents Measure: Content analysis of online forum messages	The category 'deep support' appeared to occur most frequently in the messages. In the 'deep support' category, emotional support had the highest proportion followed by 'give help'. The messages of online support communities for teenagers appeared to have a low percentage of articles e.g. a, an, the (3.74%), suggesting that the content of this discussion group was presented in a personal manner.
Stockdale & Coyne (2020). USA. High quality.	Quantitative, longitudinal, questionnaire	Setting: Longitudinal study of intrafamily life participants Sample: 385 participants that use smartphones (53% female; 17-19 years old; 70% European-American, 10% African American, 12% Multi-Ethnic,	Treatment and prevention	Social media use	Indicator(s): Empathy, social connectedness Measure(s): 7 item scale by Davis to measure empathy	Outcome: Depression and anxiety Measure: CES-DC, SCAS	All three reasons for SM use (information seeking, boredom, social connection) were positively correlated with pathological social media use and empathy (SM for social connection correlation with empathy β=.15 p=<.01). Additionally, those who start out at higher levels of using SM to

		5% Asian American, 2% Other) 70% of participants were of European-American ethnicity, Presenting condition: None in particular					connect tend to have higher levels of anxiety later in life. Using social networking sites for any reason was not related to depressive symptoms over three years.
van Rensburg, Klingensmith, McLaughlin, Qayyum, & Schalkwyk (2015). USA. High quality.	Qualitative, uncontrolled single-group design, semi-structured interviews	Setting: Yale Psychiatric Hospital Intensive Outpatient Programme Sample: 20 patients (75% female; 14-19 years old; 80% Caucasian, 15% Hispanic, 5% Mixed) Presenting condition: Combination of ADHD, Mood NOS, MDD, Anxiety, PTSD, Psychosis, ODD	Treatment	Social media for patient-provider interactions	Indicator(s): Feeling ignored, negative interactions, being able to share Mechanism(s): Easier to hide feelings, disinhibition effect, transparency, access Measure(s): Thematic analysis of interviews	Outcome: Positive and negative outcomes of patient-provider interactions via social media Measure: Thematic analysis of semi-structured interviews	Benefits: Helpful to have access to provider via digital for episodes of acute distress. Participants described a sense in which it could be easier to open up to providers about their lives and discuss sensitive issues over SM. Providers could monitor patients via Facebook status and check in on them - this would in turn reduce need for patients to explain things in therapy as provider is following on SM. Disadvantages: participants would feel anxious if they initiated communication with a provider and failed to receive a response within a short period of time. In the case of therapy, communication over SM could be a less rich experience and potentially lead to misunderstandings. Easier to hide feelings via SM, which you can't face-to-face. Provider could misunderstand posts on SM and interpret as unsafe.
van Zalk, Branje, Denissen, Van Aken, & Meeus (2011). Sweden. High quality.	Quantitative, uncontrolled single-arm longitudinal study, questionnaire	Setting: University in Utrecht Sample: 197 Psychology freshmen (78% female; mean age 18.9 years old SD=1.6; Ethnicity	Treatment and prevention	Online chatting with friends via online social networking site	Indicator(s): Social support Measure: Peer ratings	Outcome: Depression Measure: BDI Dutch short version	For the sample as a whole, chatting with online-exclusive peers and chatting with friends were not significantly associated with depression and self-esteem between measurements ($\beta<.04$, $p>.05$). In addition, this

		unknown, 92% Dutch origin) Presenting condition: None in particular					means that supportiveness did not mediate effects of chatting on emotional adjustment for the sample as a whole. For individuals with low extraversion, chatting with online exclusive peers specifically predicted less depression and more self-esteem over time.
Van Zalk & Tillfors (2017). Sweden. High quality.	Quantitative, longitudinal study, questionnaire	Setting: Swedish school Sample: 526 7th-9th graders (68% female; 13-15 years old, Ethnicity unknown, 12.1% first-generation immigrants) Presenting condition: None in particular	Treatment and prevention	Online chatting with friends via online social networking site	Indicator(s): Being able to share Measure(s): FQQ	Outcome: Depression and social anxiety Measure: CES-D, SPSQ-C	The only significant interaction that emerged was between Time-1 social anxiety and co-rumination predicting Time-2 depressive symptoms (latent unstandardized estimate =−0.13; p<.05). Co-rumination (“excessive focus on problems in close dyadic relationships with peers) buffered the link between social anxiety and depressive symptoms for adolescents with higher but not lower levels of social anxiety.
Wright et al. (2013). USA. Medium quality.	Quantitative, cross-sectional observational study, questionnaire	Setting: Undergraduate university Sample: 361 Students that use Facebook (53% female; mean age 20.26 years old SD=2.72; 77% White, 8.6% Native American, 4.4% Latino, 3.6% Asian American, 3.3% African American, 3.3% Other) Presenting condition: None in particular	Treatment and prevention	Facebook use	Indicator(s): Social support Measure: Papacharissi and Rubin's scale for measuring internet motives, Social Support Questionnaire	Outcome: Depression Measure: CES-D	Facebook social support satisfaction was negatively related to depression β=–.19 p<.01. This effect is smaller than from face-to-face social support β=-.30 p<0.01.
Yeh, Ko, Wu, & Cheng (2008). Taiwan.	Quantitative, cross-sectional, questionnaire	Setting: Project of Mental Health Survey Sample:	Treatment and prevention	Social support online	Mechanism/Indicators): Social support	Outcome: Depression Measure: Ko's	Lower actual social support and higher virtual social support were associated

Medium quality.	3477 College students (55% female; mean age 22.45 years old SD=1.56; Ethnicity unknown)<br>Presenting condition: None in particular		Mechanism(s): Conjunct face-to-face support<br>Measure(s): SSS, VSSS	Depression Inventory	with higher depressive symptoms in both gender models.

ATQ Automatic Thoughts Questionnaire BAI Beck Anxiety Inventory, BDI-II Beck's Depression Inventory II, BFNE Brief Fear of Negative Evaluation, BPRS Brief Psychiatric Rating Scale, CBT Cognitive Behavioural Therapy, CDI Children's Depression Inventory, CDRS Children's Depression Rating Scale, CES-D Center for Epidemiologic Studies Depression Scale, CF Cystic Fibrosis, CFQ-R Cognitive Failures Questionnaire, CTI Cognitive Triad Inventory, DASS Depression Anxiety Stress Scales, DSSI Duke Social Support Index, ES Effect Size, FMSS Facebook Measure of Social Support, FQQ Friendship Quality Questionnaire, HADS Hospital Anxiety and Depression Scale, ICQ Internet Communication Questionnaire INQ-15 Interpersonal Needs Questionnaire, ISSB Inventory of Socially Supportive Behaviours, LSAS Liebowitz Social Anxiety Scale, MOSSS Medical Outcomes Social Support Survey, MPPS Mothers' Perceptions of a mobile home-based Peer Support, MSPSS Multidimensional Scale of Perceived Social Support, NMMSP Networked Minds Measure of Social Presence, NRI Network of Relationships Inventory, OSSS Online Social Support Scale, PANAS Positive And Negative Affect Scale, PHQ-9 Patient Health Questionnaire Depression Scale, PSEI Peer Support Evaluation Inventory, PSSS Perceived Social Support Scale, RCMAS Revised Children's Manifest Anxiety Scale, RCT Randomised Controlled Trial, SAM Self-Assessment Manikin, SASC-R Social Anxiety Scale for Children-Revised, SCAS Spence Children’s Anxiety Scale SCS Self-Compassion Scales, SoCS Social Connectedness Scale, SCS-R Social Connectedness Scale Revised, SD standard deviation, SIAS Social Interaction Anxiety Scale, SM Social Media, SNS Social Networking Survey, SNS2 Social Network Scales, SNS3 Social Networking Survey, SPS Social Provisions Scale, SPSQ-C Social Phobia Screening Questionnaire for Children and adolescents, SSS Social Support Scale, SWEBWMS Short Warwick-Edinburgh Mental Wellbeing Scale, VSSS Virtual Social Support Scale YP Young people
