# Supplementary material for: Quality Social Connection as an Active Ingredient in Digital Interventions for Young People With Depression and Anxiety: Systematic Scoping Review and Meta-analysis
Source: J Med Internet Res. 2021 Dec 17;23(12):e26584. doi: 10.2196/26584 (PMC8726025; doi:10.2196/26584)
Supplement: Multimedia Appendix 4 [file jmir_v23i12e26584_app4.pdf]

Multimedia Appendix 4: Supplementary analyses

Psychological wellbeing

Aspects of digital QSC were examined in 10 studies focussing on psychological wellbeing outcomes [19,30-33,35,48,49,53,56]. Five studies, across six datasets, quantitatively reported change in psychological wellbeing following digital QSC (Figure S1).[30,31,33,48,58] Pooled analysis demonstrated a significant weighted mean increase in wellbeing of 10.4% (0.104, 95% CI [0.041, 0.166], p<0.0005), with high heterogeneity (I2= 84.3%).

A significant improvement in wellbeing was seen in two studies.[30, 48] Social support [30,35,56], social connectedness [31,35], loneliness (reduced) [30-32], relatedness [33], a sense of belonging [31], feeling you are not a burden [31], feeling accepted [53], connecting with similar people and emotional connection [48] were associated with improved psychological wellbeing. However, loneliness (increased) [32], negative interactions [49], and feeling ignored [49] were associated with worsened psychological wellbeing.

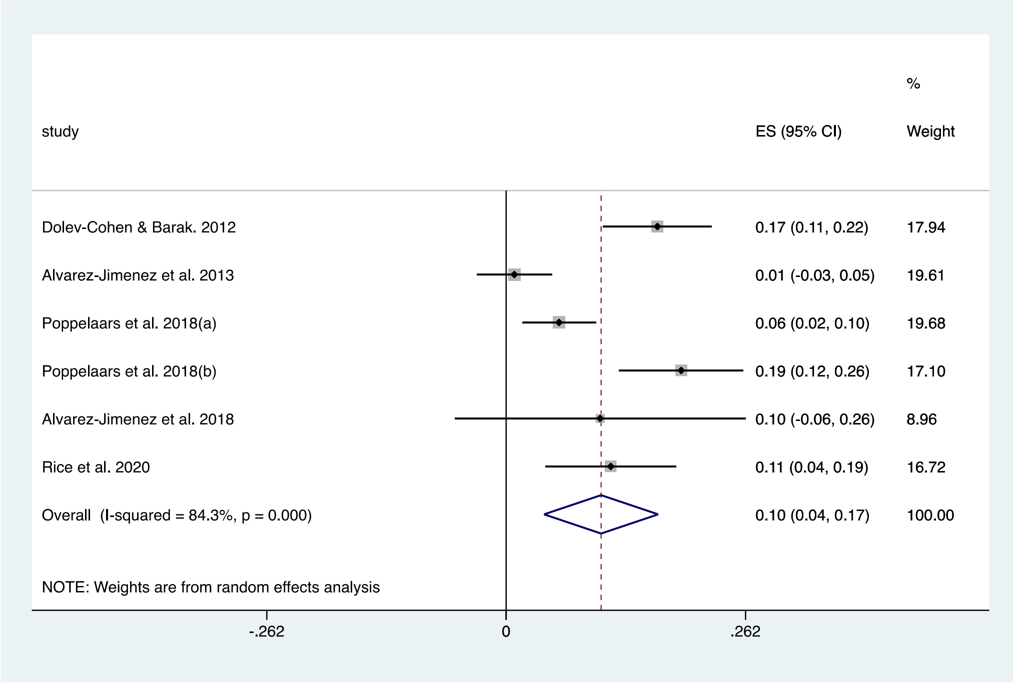

Figure S1: Forrest plot showing the effect of QSC on wellbeing outcomes
