# Supplementary material for: Quality Social Connection as an Active Ingredient in Digital Interventions for Young People With Depression and Anxiety: Systematic Scoping Review and Meta-analysis
Source: J Med Internet Res. 2021 Dec 17;23(12):e26584. doi: 10.2196/26584 (PMC8726025; doi:10.2196/26584)
Supplement: Multimedia Appendix 5 [file jmir_v23i12e26584_app5.pdf]

Professionals | 19

- Feeling safe
- Trust established
- Working towards a common goal
- Feeling heard
- Feel they can open up
- Feeling validated
- Showing empathy/establish rapport
- Feeling accepted
- Care and ability to share
- Filling the space
- Non-judgemental
- Showing understanding
- Paraphrasing
- Body language
- Transparency
- Engaged with therapy
- Provider at easer
- Intuitive feeling that it is present
- Patient feels the benefit

Young people | 19

- Feeling safe
- Trust established
- Working towards common goal
- Feeling listened to
- Active listening
- Can talk openly
- Feeling valued
- Showing empathy
- Care and ability to share
- Feeling close to someone
- Compassion
- Not feeling judged
- Feeling comfortable
- Feeling understood
- Relatedness
- Having meaningful diaglogue
- Laughing
- Being friendly
- Paying attention

Literature | 17

- Trust
- Relatedness
- Feeling normalised
- Connecting with similar people
- Empathy
- Rapport
- Feeling accepted
- Feeling close to a peer
- Being able to share
- Less alone in one's feelings
- Sense of belonging
- Shared understanding
- Emotional connection
- Feeling you are not a burden
- Feeling validated
- Social support
- Social connectedness

Indicators EXCLUDED | 7

- Social connectedness
- Social support
- Less alone in one's feelings
- Patient feels the benefit
- Provider at easer
- Engaged with therapy
- Intuitive feeling that it is present

All taken forward from expe... | 30

- Trust established (Trust established - repeat/Trust) — Also young people, Literature
- Feeling safe (feeling safe - repeat) — Also young people
- Feeling accepted (feeling accepted - repeat) — Also professionals
- Working towards a common goal (working towards common goal - repeat) — Also young people
- Relatedness (relatedness) — Literature
- Feeling heard (feeling listened to/active listening) — Also young people
- Showing empathy (showing empathy - repeat/Empathy) — Also young people, Literature
- Feeling validated (feeling valued) — Also young people, Literature
- Non-judgemental (not feeling judged) — Also young people
- Care and ability to share (being able to share) — Also young people, Literature
- Can talk openly (feel they can open up) — Also professionals
- Feeling understood (showing understanding) — Also professionals
- Feeling close to a peer (feeling close to someone) — Also young people
- Body language
- Paraphrasing
- Filling the space
- Transparency
- Compassion
- Having meaningful diaglogue
- Laughing
- Being friendly
- Paying attention
- Feeling comfortable
- Rapport
- Sense of belonging
- Shared understanding
- Emotional connection
- Feeling you are not a burden
- Connecting with similar people
- Feeling normalised

Added after feedback | 2

- Acceptance of differences
- Feeling important

Final indicators | 28

- Safety and Trust — Also professionals, Also young people
- Feeling accepted — Also professionals
- Working towards common goal — Also young people
- Relatedness — Literature
- Feeling heard
- Empathy — Also professionals, Also young people
- Feeling validated — Literature
- Non-judgemental communication — Also young people
- Sharing experiences and knowledge — Also young people, Literature
- Shared language and understanding
- Ability to be open and transparent
- Body language
- Paraphrasing
- Filling the space
- Feeling cared for
- Compassion
- Paying attention and listening effectively
- Laughing and feeling happy
- Having meaningful dialogue
- Feeling close to peer
- Feeling comfortable
- Feeling normalised
- Connecting with similar people
- Emotional connection
- Sense of belonging
- Feeling you are not a burden
- Acceptance of differences
- Feeling important
